# Targeting ABL Tyrosine Kinase in Chronic Myeloid Leukemia: Design, Synthesis, Biological Evaluation, and Computational Studies of Novel Thiazolone Derivatives

**DOI:** 10.3390/pharmaceutics18060709

**Published:** 2026-06-09

**Authors:** Belgin Sever, Halilibrahim Ciftci

**Affiliations:** 1 Department of Pharmaceutical Chemistry, Faculty of Pharmacy, Anadolu University, Eskisehir 26470, Türkiye; 2 Department of Molecular Biology and Genetics, Burdur Mehmet Akif Ersoy University, Istiklal Campus, Burdur 15200, Türkiye; 3 Medicinal and Biological Chemistry Science Farm Joint Research Laboratory, Faculty of Life Sciences, Kumamoto University, Kumamoto 862-0973, Japan

**Keywords:** chronic myeloid leukemia, ABL tyrosine kinase, thiazolone derivatives, anticancer activity, apoptosis, molecular docking

## Abstract

**Background/Objectives:** Chronic myeloid leukemia (CML) is primarily associated with the BCR:ABL1 fusion protein. Although tyrosine kinase inhibitors (TKIs) have markedly enhanced treatment outcomes, the development of agents with improved therapeutic characteristics remains necessary. The present work focused on the synthesis of a new series of thiazolone derivatives (**F1-11**) and the assessment of their anti-CML activity through inhibition of ABL tyrosine kinase (TK). **Methods:** The designed compounds were prepared through a multistep synthetic pathway involving the formation of a new chalcone intermediate (**A**), synthesis of a new pyrazoline carbothioamide intermediate (**B**), and cyclization with different aldehydes to produce the target new thiazolone derivatives (**F1-11**). Cytotoxic effects were investigated against K562 CML cells using the MTT assay. The lead compound was additionally evaluated in HL-60 AML cells and normal PBMCs. Apoptotic induction was analyzed using Annexin V/ethidium homodimer staining, whereas ABL TK inhibitory activity was measured through the ADP-Glo assay. Molecular docking studies were conducted to explore ligand interactions within the ATP-binding domain of ABL TK. **Results:** Among the synthesized molecules, **F-4** demonstrated the strongest activity against K562 cells with an IC_50_ value of 6.85 µM, close to that observed for imatinib (IC_50_ = 5.20 µM). The compound showed reduced cytotoxicity toward HL-60 cells (IC_50_ = 33.44 µM) and exhibited favorable selectivity toward PBMCs (SI = 13). Apoptosis studies revealed 51% early apoptotic cells and 43% late apoptotic cells following treatment. In the kinase assay, **F-4** inhibited ABL TK activity by 39% at 10 µM and by 70% at 100 µM. Docking simulations suggested interactions with residues His361 and Asp381 in addition to nearby hydrophobic amino acids, although the interaction network was less extensive than that of imatinib. **Conclusions:** The findings identify **F-4** as a promising new thiazolone-derived scaffold with selective anti-CML activity and notable ABL TK inhibitory potential. Additional structural optimization may further enhance its binding characteristics and therapeutic efficacy.

## 1. Introduction

Chronic myeloid leukemia (CML) is a clonal hematological malignancy originating from the transformation of hematopoietic stem cells, leading to the excessive accumulation of myeloid lineage cells in the bone marrow and peripheral circulation [1,2,3]. The disease is defined at the molecular level by the presence of the Philadelphia chromosome, which arises from a reciprocal translocation between chromosomes 9 and 22 and results in the formation of the BCR-ABL1 fusion gene [4,5,6,7]. The encoded BCR-ABL1 oncoprotein exhibits constitutive tyrosine kinase (TK) activity, which disrupts normal cellular signaling and promotes uncontrolled proliferation while suppressing apoptotic pathways [8,9]. Clinically, CML progresses through chronic, accelerated, and blast phases, reflecting increasing genomic instability and disease severity [4,10,11].

At the molecular level, the oncogenic activity of BCR-ABL1 is primarily attributed to its deregulated TK function. Unlike the tightly controlled native ABL TK, the fusion protein remains constitutively active due to the loss of normal regulatory mechanisms [12,13]. This persistent activation results in continuous stimulation of multiple downstream signaling pathways, including PI3K/AKT, RAS/MAPK, and JAK/STAT, which collectively enhance cell survival, proliferation, and resistance to apoptosis [14,15,16,17,18,19]. In addition, BCR-ABL1 has been implicated in genomic instability and impaired DNA repair processes, contributing to disease progression and therapeutic resistance [8,18]. The persistence of leukemic stem cells, which are relatively insensitive to therapy, further complicates treatment and plays a critical role in minimal residual disease and relapse [20,21,22].

**Figure 1:**
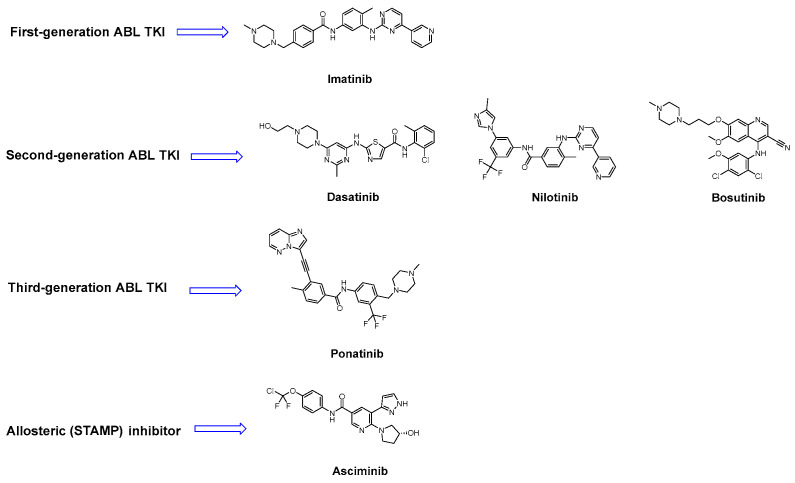
Representative structures of first-, second-, and third-generation ABL TKIs with STAMP inhibitor asciminib.

The development of BCR-ABL1-targeted tyrosine kinase inhibitors (TKIs) has markedly improved the clinical management of CML. Imatinib, the first-generation TKI, selectively binds to the ATP-binding site of the TK and has significantly improved survival outcomes, effectively transforming CML into a manageable chronic condition [23,24,25,26,27]. However, the emergence of resistance and suboptimal responses in some patients has led to the development of second-generation TKIs. Among these, dasatinib has demonstrated markedly enhanced inhibitory activity and strong clinical efficacy in BCR-ABL1-positive leukemias [28,29,30], while nilotinib exhibits improved binding affinity and potency. Bosutinib, which functions as a dual Src/ABL TK inhibitor, provides an alternative targeting profile [31]. In addition, ponatinib, a third-generation TKI, was specifically developed to overcome resistant mutations, including the clinically significant T315I substitution [32,33]. More recently, asciminib has been introduced as a first-in-class allosteric inhibitor that selectively targets the myristoyl-binding pocket of ABL TK, offering a mechanism distinct from conventional ATP-competitive inhibitors (Figure 1) [12,34].

Despite these advances, resistance to TKIs remains a major clinical challenge. Mutations within the TK domain, activation of alternative signaling pathways, and microenvironmental influences contribute to reduced drug sensitivity and disease persistence [35,36,37]. In particular, mutations such as T315I significantly impair the efficacy of many ATP-competitive inhibitors [33]. Furthermore, leukemic stem cells can survive independently of ABL TK inhibition, leading to relapse even after an initial therapeutic response [19,21]. Long-term TKI therapy is also associated with adverse effects and tolerability issues, which may require dose adjustments or therapy modifications [21,38].

Recent advances in molecular diagnostics and next-generation sequencing technologies have enhanced the understanding of disease heterogeneity, clonal evolution, and resistance mechanisms, enabling the development of more personalized therapeutic approaches [13,39,40]. Moreover, novel therapeutic strategies, including allosteric inhibition, targeted protein degradation, and rational medicinal chemistry approaches, are being actively explored to overcome current treatment limitations [41,42,43]. Collectively, these developments highlight the ongoing need for the discovery and optimization of new small-molecule ABL TKIs with improved efficacy, selectivity, and safety profiles for the treatment of CML.

**Figure 2:**
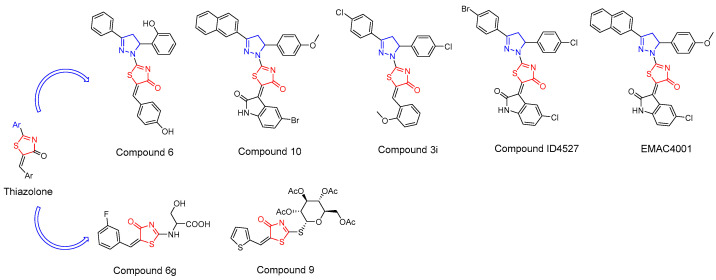
Thiazolone derivatives with anticancer activity.

Thiazole is a five-membered heterocyclic ring system incorporating both an electron-donating sulfur atom and an electron-withdrawing nitrogen atom (–C=N), which together contribute to its unique electronic properties. Its aromatic character arises from the delocalization of lone pair electrons, particularly those associated with the sulfur atom [44,45,46,47,48]. Thiazolone derivatives, representing an important subclass of thiazoles, have attracted considerable interest due to their wide range of biological activities, especially their notable anticancer potential [49,50,51,52,53,54,55,56]. Several studies have reported highly active compounds within this class; for instance, compounds 6 [50], 10 [51], 3i [52], ID4527 [53], EMAC4001 [54], 6g [55], and 9 [56] exhibited pronounced cytotoxic effects against various cancer cell lines (Figure 2). In medicinal chemistry, the thiazolone scaffold is frequently combined with other heterocyclic systems as a strategy to optimize pharmacological profiles. Such hybridization approaches often result in enhanced biological activity, improved selectivity, and reduced toxicity. In particular, hybrid structures combining pyrazoline and thiazolone moieties, as illustrated by compounds 6, 10, 3i, ID4527, and EMAC4001 (Figure 2), have been recognized as promising scaffolds for the development of kinase-targeted anticancer agents [50,51,52,53,54].

In this work, a series of novel thiazolone derivatives (**F1-11**) was developed through a stepwise synthetic route starting from the new chalcone (**A**), followed by conversion into the corresponding new pyrazoline carbothioamide (**B**), and subsequent cyclization to yield the target thiazolones. The antiproliferative potential of **F1-11** was initially screened against K562 CML cells using the MTT assay. Based on these results, the most active compound was further evaluated in HL-60 acute myeloid leukemia (AML) cells as well as in healthy peripheral blood mononuclear cells (PBMCs) to assess its selectivity profile. To gain insight into its mode of action, apoptosis induction in K562 cells was analyzed using Annexin V/ethidium homodimer staining. In parallel, the inhibitory effect on ABL TK activity was determined via the ADP-Glo kinase assay. Additionally, molecular docking studies were performed using the Maestro platform to investigate the binding mode of the active compound within the ATP-binding pocket of ABL TK.

## 2. Materials and Methods

### 2.1. Chemistry

All chemicals and reagents were obtained from commercial suppliers and were used as received, unless otherwise noted. Reaction progress was monitored by thin-layer chromatography (TLC) using silica gel 60 F254 aluminum plates (Merck, Darmstadt, Germany). ^1^H and ^13^C nuclear magnetic resonance (NMR) spectra were acquired on a Bruker spectrometer (Bruker, Billerica, MA, USA). High-resolution mass spectrometric (HRMS) analyses were carried out using a JEOL JMS-700 Station/JMS-BU-20-Gcmate system (JEOL, Akishima, Tokyo, Japan) for compounds **A** and **B**, and **F-1**, while the **F1-11** derivatives were analyzed using an Agilent 6530 Accurate-Mass Q-TOF LC/MS instrument (Agilent Technologies, Santa Clara, CA, USA).

#### 2.1.1. Synthesis of (E)-3-(5-(4-Chlorophenyl)furan-2-yl)-1-(3,4-dimethylphenyl)prop-2-en-1-one (**A**)

1-(3,4-Dimethylphenyl)ethan-1-one (1 mmol) and 5-(4-chlorophenyl)furan-2-carbaldehyde (1 mmol) were dissolved in ethanol, followed by the addition of NaOH (1.1 mmol). The reaction mixture was stirred at ambient temperature for 24 h. The progress of the reaction was monitored by TLC. After completion, the reaction mixture was poured into crushed ice to facilitate precipitation. The resulting precipitate was collected by filtration, washed thoroughly with water, and air-dried. The crude product was subsequently purified by recrystallization from ethanol [57,58,59].

Compound **A**: Yellow solid. Yield: 82%. M.p. 142–143 °C. ^1^H NMR (500 MHz, *CDCl*_3_) *δ* (ppm): 2.34 (3H, s, CH_3_), 2.36 (3H, s, CH_3_), 6.76 (2H, d, *J* = 12.3 Hz), 7.26 (1H, d, *J* = 7.2 Hz), 7.40 (2H, d, *J* = 9.0 Hz), 7.49 (1H, d, *J* = 15.5 Hz), 7.58 (1H, d, *J* = 15.2 Hz), 7.69 (2H, d, *J* = 8.4 Hz), 7.79 (1H, d, *J* = 7.3 Hz), 7.83 (1H, s). ^13^C NMR (125 MHz, *CDCl*_3_) *δ* (ppm): 19.6 (CH_3_), 20.1 (CH_3_), 108.7 (CH), 118.6 (CH), 119.6 (CH), 125.7 (2CH), 126.2 (CH), 128.4 (C), 129.2 (2CH), 129.6 (CH), 129.8 (CH), 129.9 (CH), 134.3 (C), 136.1 (C), 137.1 (C), 142.5 (C), 151.6 (C), 155.1 (C), 189.6 (C, C=O). HRMS (FAB) calcd. For C_21_H_17_ClO_2_ [M+H]^+^: *m*/*z* = 337.0995; found: 337.0989. (Spectral Data: Supplementary Information. Figures S1–S3).

#### 2.1.2. Synthesis of 5-(5-(4-Chlorophenyl)furan-2-yl)-3-(3,4-dimethylphenyl)-4,5-dihydro-1H-pyrazole-1-carbothioamide (**B**)

Compound **A** (3 mmol) was reacted with thiosemicarbazide (4.5 mmol) in the presence of NaOH (3 mmol) in ethanol under reflux conditions for 8–12 h. The progress of the reaction was monitored by TLC. Upon completion, the reaction mixture was poured into crushed ice and allowed to cool. The formed precipitate was collected by filtration, washed with water, and dried. The crude product was further purified by recrystallization from ethanol [57,58,59].

Compound **B**: Light brown solid. Yield: 80%. M.p. 179–180 °C. ^1^H NMR (500 MHz, *CDCl*_3_) *δ* (ppm): *δ* (ppm): 2.32 (6H, s), 3.51 (1H, dd, *J_AB_* = 17.9 Hz, *J_AX_* = 3.3 Hz), 3.69 (1H, dd, *J_BA_* = 17.7 Hz, *J_BX_* = 11.5 Hz), 6.16 (1H, dd, *J_BX_* = 11.3 Hz, *J_AX_* = 3.6 Hz), 6.48 (1H, s), 6.56 (1H, s), 7.21 (1H, d, *J* = 7.9 Hz), 7.27 (2H, d, *J* = 8.4 Hz), 7.45–7.49 (3H, m), 7.56 (1H, s). ^13^C NMR (125 MHz, *CDCl*_3_) *δ* (ppm): 19.7 (2CH_3_), 39.9 (CH_2_), 56.9 (CH), 106.1 (CH), 110.9 (CH), 124.7 (CH), 125.1 (2CH), 128.0 (C), 128.7 (2CH), 129.2 (C), 130.1 (CH), 132.9 (CH), 129.9 (CH), 134.3 (C), 137.5 (C), 140.5 (C), 151.7 (C), 152.5 (C), 156.6 (C), 176.4 (C). HRMS (FAB) calcd. For C_22_H_20_ClN_3_OS [M+H]^+^: *m*/*z* = 410.1094; found: 410.1081. (Spectral Data: Supplementary Information. Figures S4–S6).

#### 2.1.3. Synthesis of (Z)-2-(5-(5-(4-Chlorophenyl)furan-2-yl)-3-(3,4-dimethylphenyl)-4,5-dihydro-1H-pyrazol-1-yl)-5-(aryl)methylene)thiazol-4(5H)-one (**F1-11**)

Compound **B** (10 mmol), chloroacetic acid (10 mmol), the appropriate aldehyde (10 mmol), and anhydrous sodium acetate (10 mmol) were stirred in glacial acetic acid (10 mL) under reflux for 5 h. After cooling, the resulting solid was filtered, washed with methanol, and recrystallized from DMF:ethanol or DMF:acetic acid mixtures (1:2, *v*/*v*) [50].

(Z)-2-(5-(5-(4-Chlorophenyl)furan-2-yl)-3-(3,4-dimethylphenyl)-4,5-dihydro-1H-pyrazol-1-yl)-5-(5-chloropyridin-2-yl)methylene)thiazol-4(5H)-one (**F-1**)**:** Light brown solid. Yield: 75%. M.p. 158–159 °C. ^1^H NMR (500 MHz, *CDCl*_3_) *δ* (ppm): 2.34 (6H, s), 3.70 (1H, dd, *J_AB_* = 17.6 Hz, *J_AX_* = 4.0 Hz), 3.81 (1H, dd, *J_BA_* = 17.7 Hz, *J_BX_* = 11.1 Hz), 5.91 (1H, dd, *J_BX_* = 11.3 Hz, *J_AX_* = 4.4 Hz), 6.55 (1H, d, *J* = 4.6 Hz), 6.58 (1H, d, *J* = 3.3 Hz), 6.62–6.66 (1H, m), 7.23–7.27 (4H, m), 7.41–7.44 (3H, m), 7.54 (1H, d, *J* = 9.8 Hz), 7.53–7.68 (2H, m). ^13^C NMR (125 MHz, *CDCl*_3_) *δ* (ppm): 19.7 (2CH_3_), 39.6 (CH_2_), 56.9 (CH), 105.9 (CH), 111.7 (CH), 124.9 (CH), 125.0 (3CH), 127.4 (C), 128.3 (CH), 128.7 (CH), 128.9 (4CH), 130.0 (C), 133.8 (2C), 137.1 (2C), 140.9 (2C), 149.3 (2C), 153.1 (CH), 159.9 (C), 178.3 (C), 187.9 (C). HRMS (FAB) calcd. For C_30_H_22_Cl_2_N_4_O_2_S [M+H]^+^: (*m*/*z*) = 573.0919; found: 573.0901. (Spectral Data: Supplementary Information. Figures S7–S9).

(Z)-2-(5-(5-(4-Chlorophenyl)furan-2-yl)-3-(3,4-dimethylphenyl)-4,5-dihydro-1H-pyrazol-1-yl)-5-(benzo[d][1,3]dioxol-5-ylmethylene)thiazol-4(5H)-one (**F-2**): Light Brown solid. Yield: 70%. M.p. 184–185 °C. ^1^H NMR (500 MHz, *CDCl*_3_) *δ* (ppm): 2.34 (6H, s), 3.71 (1H, dd, *J_AB_* = 17.6 Hz, *J_AX_* = 4.7 Hz), 3.80 (1H, dd, *J_BA_* = 17.8 Hz, *J_BX_* = 11.8 Hz), 5.88 (1H, dd, *J_BX_* = 10.8 Hz, *J_AX_* = 4.1 Hz), 6.04 and 6.07 (2H, 2s), 6.55 (1H, d, *J* = 3.5 Hz), 6.59 (1H, d, *J* = 3.9 Hz), 7.23–7.28 (m, 6H), 7.44 (3H, d, *J* = 8.0 Hz), 7.59 (2H, d, *J* = 7.6 Hz). ^13^C NMR (125 MHz, *CDCl*_3_) *δ* (ppm): 20.0 (2CH_3_), 39.2 (CH_2_), 57.6 (CH, C_5_), 102.1 (CH_2_), 106.4 (CH), 108.4 (CH), 112.2 (2CH), 123.9 (CH), 124.9 (CH), 125.1 (2CH), 127.5 (C), 128.4 (2CH), 128.7 (C), 128.8 (2CH), 130.1 (2C), 133.6 (C), 133.7 (C), 141.3 (C), 149.0 (2C), 149.6 (2C), 153.4 (CH), 160.0 (C), 178.3 (C), 188.1 (C). HRMS (ESI) calcd. For C_32_H_24_ClN_3_O_4_S [M+H]^+^: (*m*/*z*) = 581.1176; found: 581.11962. (Spectral Data: Supplementary Information. Figures S10–S12).

(Z)-2-(5-(5-(4-Chlorophenyl)furan-2-yl)-3-(3,4-dimethylphenyl)-4,5-dihydro-1H-pyrazol-1-yl)-5-((5-bromobenzo[d][1,3]dioxol-4-yl)methylene)thiazol-4(5H)-one (**F-3**): Yellow solid. Yield: 72%. M.p. 182–183 °C. ^1^H NMR (500 MHz, *CDCl*_3_) *δ* (ppm): 2.33 (6H, s), 3.69–3.73 (1H, m), 3.80–3.83 (1H, m), 5.88 (1H, dd, *J_BX_* = 11.3 Hz, *J_AX_* = 4.8 Hz), 6.12 (1H, d, *J* = 6.5 Hz), 6.16 (2H, s), 6.55–6.56 (1H, m), 6.59 (1H, d, *J* = 3.3 Hz), 6.84 (1H, d, *J* = 8.3 Hz), 7.09 (1H, d, *J* = 8.6 Hz), 7.23–7.27 (3H, m), 7.43–7.45 (2H, m), 7.54–7.57 (1H, m), 7.61 (1H, d, *J* = 8.9 Hz). ^13^C NMR (125 MHz, *CDCl*_3_) *δ* (ppm): 20.1 (2CH_3_), 39.3 (CH_2_), 57.5 (CH), 103.9 (CH_2_), 106.5 (CH), 109.8 (CH), 113.9 (CH), 124.9 (2CH), 125.7 (CH), 126.2 (2CH), 127.4 (C), 128.2 (CH), 129.0 (2CH), 130.5 (2C), 133.4 (2C), 134.1 (C), 137.3 (C), 141.2 (C), 147.5 (C), 148.8 (C), 149.3 (C), 149.5 (C), 153.1 (CH), 159.9 (C), 171.6 (C), 179.9 (C). HRMS (ESI) calcd. For C_32_H_23_BrClN_3_O_4_S [M+H]^+^: *m*/*z* = 659.02812; found: 659.0305. (Spectral Data: Supplementary Information. Figures S13–S15).

(Z)-2-(5-(5-(4-Chlorophenyl)furan-2-yl)-3-(3,4-dimethylphenyl)-4,5-dihydro-1H-pyrazol-1-yl)-5-((6-chloropyridin-3-yl)methylene)thiazol-4(5H)-one (**F-4**): Light brown solid. Yield: 74%. M.p. 167–168 °C. ^1^H NMR (500 MHz, DMSO-*d_6_*) *δ* (ppm): 2.28 (3H, s), 2.30 (3H, s), 3.72–3.82 (1H, m), 3.99–4.07 (1H, m), 5.92 (1H, dd, *J_BX_* = 11.2 Hz, *J_AX_* = 4.3 Hz), 6.51 (1H, d, *J* = 8.9 Hz), 6.60 (1H, d, *J* = 3.5 Hz), 6.67 (1H, d, *J* = 3.9 Hz), 6.95–6.99 (2H, m), 7.30 (2H, d, *J* = 7.7 Hz), 7.42–7.44 (3H, m), 7.60–7.62 (3H, m). ^13^C NMR (125 MHz, *CDCl*_3_) *δ* (ppm): 19.7 (2CH_3_), 46.3 (CH_2_), 57.5 (CH), 107.5 (CH), 111.5 (CH), 123.2 (CH), 124.5 (CH), 125.2 (2CH), 127.0 (C), 127.2 (C), 127.9 (2CH), 128.7 (CH), 129.1 (2CH), 130.1 (C), 132.1 (C), 136.8 (CH), 137.1 (C), 140.0 (C), 140.5 (CH), 150.5 (C), 150.9 (C), 151.7 (CH), 151.9 (C), 160.6 (C), 169.6 (C), 177.5 (C), 186.9 (C). HRMS (ESI) calcd. For C_22_H_22_Cl_2_N_4_O_2_S [M+H]^+^: *m*/*z* = 572.08405; found: 572.08455. (Spectral Data: Supplementary Information. Figures S16–S18).

(Z)-2-(5-(5-(4-Chlorophenyl)furan-2-yl)-3-(3,4-dimethylphenyl)-4,5-dihydro-1H-pyrazol-1-yl)-5-((1H-indol-3-yl)methylene)thiazol-4(5H)-one (**F-5**): Yellow solid. Yield: 76%. M.p. 213–214 °C. ^1^H NMR (500 MHz, *CDCl*_3_) *δ* (ppm): 2.26 (3H, s), 2.32 (3H, s), 3.58–3.63 (1H, m), 3.67–3.78 (1H, m), 5.82 (1H, dd, *J_BX_* = 11.1 Hz, *J_AX_* = 4.0 Hz), 6.43–6.49 (2H, m), 7.13–7.26 (4H, m), 7.35 (1H, d, *J* = 7.1 Hz), 7.40 (1H, d, *J* = 9.5 Hz), 7.44–7.52 (3H, m), 7.57 (1H, d, *J* = 10.5 Hz), 7.63–7.72 (2H, m), 8.10 (1H, s), 10.28 (1H, s). ^13^C NMR (125 MHz, *CDCl*_3_) *δ* (ppm): 20.0 (2CH_3_), 39.5 (CH_2_), 57.1 (CH), 106.5 (CH), 109.7 (CH), 111.8 (CH), 112.2 (C), 114.2 (CH), 118.7 (CH), 121.2 (CH), 122.2 (CH), 123.2 (C), 125.0 (2CH), 126.1 (CH), 127.4 (C), 128.3 (C), 128.8 (2CH), 128.9 (2CH), 130.2 (C), 133.2 (C), 136.4 (C), 137.5 (C), 141.1 (C), 149.3 (C), 149.6 (C), 152.9 (CH), 159.7 (C), 181.7 (C), 188.2 (C). HRMS (ESI) calcd. For C_33_H_25_ClN_4_O_2_S [M+H]^+^: *m*/*z* = 576.13867; found: 576.1405. (Spectral Data: Supplementary Information. Figures S19–S21).

(Z)-2-(5-(5-(4-Chlorophenyl)furan-2-yl)-3-(3,4-dimethylphenyl)-4,5-dihydro-1H-pyrazol-1-yl)-5-(thiophen-2-ylmethylene)thiazol-4(5H)-one (**F-6**): Light brown solid. Yield: 75%. M.p. 160–161 °C. ^1^H NMR (500 MHz, *CDCl*_3_) *δ* (ppm): 22.33 (6H, s), 3.69 (1H, dd, *J_AB_* = 17.7 Hz, *J_AX_* = 4.5 Hz), 3.75–3.82 (1H, m), 5.87 (1H, dd, *J_BX_* = 10.9 Hz, *J_AX_* = 4.3 Hz), 6.54 (1H, d, *J* = 3.4 Hz), 6.58 (1H, d, *J* = 3.7 Hz), 7.22–7.26 (5H, m), 7.43 (3H, d, *J* = 10.2 Hz), 7.54 (1H, d, *J* = 8.7 Hz), 7.61–7.66 (2H, m). ^13^C NMR (125 MHz, *CDCl*_3_) *δ* (ppm): 19.7 (2CH_3_), 38.9 (CH_2_), 57.3 (CH), 106.5 (CH), 112.1 (CH), 124.9 (CH), 125.1 (2CH), 127.4 (C), 128.3 (CH), 128.8 (CH), 128.9 (4CH), 130.0 (CH), 130.2 (2C), 133.3 (C), 137.5 (2C), 141.2 (C), 149.5 (2C), 153.0 (CH), 160.0 (C), 178.2 (C), 187.8 (C). HRMS (ESI) calcd. For C_29_H_22_ClN_3_O_2_S_2_ [M+H]^+^: *m*/*z* = 543.0842; found: 543.08669. (Spectral Data: Supplementary Information. Figures S22–S24).

(Z)-2-(5-(5-(4-Chlorophenyl)furan-2-yl)-3-(3,4-dimethylphenyl)-4,5-dihydro-1H-pyrazol-1-yl)-5-((5-methylthiophen-2-yl)methylene)thiazol-4(5H)-one (**F-7**): Light brown solid. Yield: 70%. M.p. 169–170 °C. ^1^H NMR (500 MHz, *CDCl*_3_) *δ* (ppm): 2.33 (9H, s), 3.70 (1H, dd, *J_AB_* = 17.4 Hz, *J_AX_* = 4.0 Hz), 3.80 (1H, dd, *J_BA_* = 17.6 Hz, *J_BX_* = 10.8 Hz), 5.87 (1H, dd, *J_BX_* = 10.9 Hz, *J_AX_* = 4.2 Hz), 6.54 (1H, d, *J* = 3.4 Hz), 6.59 (1H, d, *J* = 3.6 Hz), 7.23–7.27 (5H, m), 7.44 (3H, d, *J* = 10.2 Hz), 7.54 (1H, d, *J* = 8.4 Hz), 7.59–7.66 (1H, m). ^13^C NMR (125 MHz, *CDCl*_3_) *δ* (ppm): 20.8 (3CH_3_), 39.5 (CH_2_), 57.5 (CH), 106.4 (CH), 112.0 (CH), 124.9 (CH), 125.1 (2CH), 127.4 (C), 128.3 (C), 128.8 (CH), 128.9 (4CH), 130.3 (2C), 133.3 (CH), 137.5 (2C), 141.2 (2C), 149.4 (2C), 152.9 (CH), 160.0 (C), 178.2 (C), 187.8 (C). HRMS (ESI) calcd. For C_30_H_24_ClN_3_O_2_S_2_ [M+H]^+^: *m*/*z* = 557.10244; found: 557.09985. (Spectral Data: Supplementary Information. Figures S25–S27).

(Z)-2-(5-(5-(4-chlorophenyl)furan-2-yl)-3-(3,4-dimethylphenyl)-4,5-dihydro-1H-pyrazol-1-yl)-5-(furan-2-ylmethylene)thiazol-4(5H)-one (**F-8**): Light brown solid. Yield: 78%. M.p. 163–164 °C. ^1^H NMR (500 MHz, *CDCl*_3_) *δ* (ppm): 2.33 (6H, s), 3.70 (1H, dd, *J_AB_* = 17.1 Hz, *J_AX_* = 4.6 Hz), 3.74–3.82 (1H, m), 5.87 (1H, dd, *J_BX_* = 11.3 Hz, *J_AX_* = 4.4 Hz), 6.53–6.55 (1H, m), 6.59 (1H, d, *J* = 5.2 Hz), 6.62 (1H, d, *J* = 5.1 Hz), 6.69 (1H, d, *J* = 4.1 Hz), 7.23–7.27 (2H, m), 7.44 (1H, d, *J* = 8.7 Hz), 7.53–7.55 (2H, m), 7.58–7.65 (4H, m). ^13^C NMR (125 MHz, *CDCl*_3_) *δ* (ppm): 19.8 (2CH_3_), 39.5 (CH_2_), 57.9 (CH), 106.8 (CH), 112.1 (CH), 116.1 (CH), 118.4 (CH), 124.9 (CH), 125.1 (2CH), 128.2 (C), 128.8 (C), 128.9 (2CH), 130.3 (2CH), 133.3 (C), 137.5 (2C), 141.2 (C), 145.4 (CH), 149.4 (2C), 150.6 (C), 153.1 (CH), 160.0 (C), 180.6 (C), 187.8 (C). HRMS (ESI) calcd. For C_29_H_22_ClN_3_O_3_S [M+H]^+^: (*m*/*z*) = 527.10704; found: 527.10875. (Spectral Data: Supplementary Information. Figures S28–S30).

(Z)-2-(5-(5-(4-Chlorophenyl)furan-2-yl)-3-(3,4-dimethylphenyl)-4,5-dihydro-1H-pyrazol-1-yl)-5-((5-methylfuran-2-yl)methylene)thiazol-4(5H)-one (**F-9**): Yellow solid. Yield: 80%. M.p. 172–173 °C. ^1^H NMR (500 MHz, *CDCl*_3_) *δ* (ppm): 2.34 (3H, s), 2.35 (3H, s), 2.36 (3H, s), 3.73 (1H, dd, *J_AB_* = 17.3 Hz, *J_AX_* = 4.1 Hz), 3.79–3.83 (1H, m), 5.87 (1H, dd, *J_BX_* = 11.0 Hz, *J_AX_* = 4.6 Hz), 6.54–6.55 (2H, m), 6.59 (1H, d, *J* = 3.5 Hz), 6.61 (1H, d, *J* = 3.3 Hz), 7.25–7.27 (2H, m), 7.43–7.46 (2H, m), 7.45 (1H, s), 7.54 (1H, d, *J* = 8.2 Hz), 7.59–7.64 (2H, m). ^13^C NMR (125 MHz, *CDCl*_3_) *δ* (ppm): 14.7 (CH_3_), 19.9 (2CH_3_), 39.8 (CH_2_), 57.1 (CH), 106.5 (CH), 109.5 (CH), 111.9 (CH), 117.8 (CH), 124.2 (CH), 124.9 (2CH), 127.5 (C), 128.2 (C), 128.8 (2CH), 130.3 (2CH), 133.2 (C), 137.6 (2C), 141.1 (C), 149.1 (C), 149.4 (C), 149.7 (C), 153.0 (CH), 156.4 (C), 160.0 (C), 178.2 (C), 187.6 (C). HRMS (ESI) calcd. For C_30_H_24_ClN_3_O_3_S [M+H]^+^: *m*/*z* = 541.12269; found: 541.12447. (Spectral Data: Supplementary Information. Figures S31–S33).

(Z)-2-(5-(5-(4-Chlorophenyl)furan-2-yl)-3-(3,4-dimethylphenyl)-4,5-dihydro-1H-pyrazol-1-yl)-5-((5-(4-bromophenyl)furan-2-yl)methylene)-thiazol-4(5H)-one (**F-10**): Yellow solid. Yield: 79%. M.p. 229–230 °C. ^1^H NMR (500 MHz, *CDCl*_3_) *δ* (ppm): 2.34 (6H, s), 3.72–3.74 (1H, m), 3.79–3.83 (1H, m), 5.97 (1H, dd, *J_BX_* = 11.8 Hz, *J_AX_* = 4.2 Hz), 6.59 (1H, d, *J* = 4.6 Hz), 6.64 (1H, d, *J* = 4.2 Hz), 6.76 (1H, d, *J* = 4.6 Hz), 6.79 (1H, d, *J* = 4.3 Hz), 7.24–7.31 (6H, m), 7.44 (2H, d, *J* = 8.5 Hz), 7.55–7.58 (2H, m), 7.66–7.68 (2H, m). ^13^C NMR (125 MHz, *CDCl*_3_) *δ* (ppm): 20.1 (2CH_3_), 39.4 (CH_2_), 57.3 (CH), 106.5 (CH), 108.0 (CH), 112.3 (CH), 117.9 (CH), 122.5 (CH), 123.9 (2CH), 125.2 (2CH), 125.9 (C), 126.7 (2CH), 128.1 (2C), 130.5 (C), 132.4 (4CH), 133.3 (C), 137.5 (2C), 141.1 (C), 149.5 (C), 150.3 (C), 152.2 (C), 153.2 (C), 155.5 (CH), 158.3 (C), 177.4 (C), 187.8 (C). HRMS (ESI) calcd. For C_35_H_25_BrClN_3_O_3_S [M+H]^+^: *m*/*z* = 681.04885; found: 681.05073. (Spectral Data: Supplementary Information. Figures S34–S36).

(Z)-2-(5-(5-(4-Chlorophenyl)furan-2-yl)-3-(3,4-dimethylphenyl)-4,5-dihydro-1H-pyrazol-1-yl)-5-(4-chlorobenzylidene)thiazol-4(5H)-one (**F-11**): Light brown solid. Yield: 76%. M.p. 188–189 °C. ^1^H NMR (500 MHz, DMSO-*d_6_*) *δ* (ppm): 2.34 (6H, s), 3.68–3.74 (1H, m), 3.77–3.83 (1H, m), 5.88 (1H, dd, *J_BX_* = 11.1 Hz, *J_AX_* = 4.4 Hz), 6.55 (2H, t, *J* = 3.8 Hz), 6.59 (1H, d, *J* = 3.8 Hz), 6.64 (1H, d, *J* = 3.7 Hz), 7.23–7.24 (2H, m), 7.40–7.44 (2H, m), 7.46–7.49 (2H, m), 7.54–7.61 (2H, m), 7.65 (1H, s), 7.71 (1H, s). ^13^C NMR (125 MHz, *CDCl*_3_) *δ* (ppm): 19.9 (2CH_3_), 39.8 (CH_2_), 57.7 (CH), 106.7 (CH), 112.3 (CH), 124.9 (CH), 125.3 (2CH), 127.4 (CH), 128.7 (C), 128.9 (4CH), 129.3 (2CH), 130.3 (C), 130.8 (C), 132.9 (C), 133.4 (C), 135.6 (C), 137.7 (C), 141.5 (C), 149.2 (C), 149.5 (C), 153.1 (CH), 160.0 (C), 178.2 (C), 188.3 (C). HRMS (ESI) calcd. For C_31_H_23_Cl_2_N_3_O_2_S [M+H]^+^: *m*/*z* = 571.0888; found: 571.09048. (Spectral Data: Supplementary Information. Figures S37–S39).

### 2.2. Biological Evaluation

#### 2.2.1. Cytotoxicity Assay

K562 and HL-60 leukemia cell lines (ATCC, Manassas, VA, USA), together with human peripheral blood mononuclear cells (PBMCs) supplied by Precision Bioservices (Frederick, MD, USA), were cultured in RPMI-1640 medium obtained from Wako Pure Chemical Industries. The medium was enriched with 5% fetal bovine serum (Sigma-Aldrich, St. Louis, MO, USA) and streptomycin (89 μg/mL; Meiji Seika Pharma, Tokyo, Japan). Cell cultures were maintained at 37 °C under humidified conditions with 5% CO_2_. During the logarithmic growth phase, K562 and HL-60 cells were seeded at 4 × 10^4^ cells/mL, whereas PBMCs were plated at 1 × 10^6^ cells/mL. The synthesized derivatives were then added, and the cells were incubated for 48 h. Test compounds and imatinib were initially dissolved in DMSO and subsequently diluted with culture medium to the required concentrations while keeping the final DMSO concentration below 1% [60,61,62].

Cell survival was assessed using the MTT colorimetric method [63], based on the conversion of MTT into insoluble formazan by viable cells. After 48 h of exposure to the compounds, MTT reagent was introduced, and incubation continued for 4 h at 37 °C. The culture medium was discarded, and the resulting formazan crystals were solubilized in DMSO. Optical density was measured at 570 nm using a Tecan Infinite M1000 microplate reader (Austria). Experiments were performed in triplicate, and IC_50_ values were defined as the concentration causing a 50% reduction in cell viability. Imatinib served as the positive control [60,61,62].

#### 2.2.2. Apoptosis Assay

The apoptotic effect of **F-4** was examined in K562 cells following treatment with the compound at its IC_50_ value for 12 h. After treatment, cells were harvested and rinsed using binding buffer. Samples were subsequently stained for 20 min in the dark at room temperature with a solution containing FITC-Annexin V, ethidium homodimer III, and Hoechst 33342. Following staining, the cells were washed, fixed using paraformaldehyde, and finally rinsed with phosphate-buffered saline (PBS). Fluorescence imaging was carried out using a Biorevo BZ-9000 microscope (Keyence, Osaka, Japan) to differentiate apoptotic from necrotic cell populations [60,61,62].

#### 2.2.3. ABL TK Inhibition Assay

The ability of **F-4** to inhibit ABL TK was investigated in comparison with imatinib using the Promega kinase assay kit (V1901). Assays were performed in reaction buffer containing Tris (pH 7.5), MgCl_2_, bovine serum albumin, ATP, and DTT. Reactions were assembled in 384-well plates by mixing enzyme preparation, substrate solution, and varying concentrations of the tested compounds. Following a 2 h incubation period at ambient temperature, kinase activity was evaluated through the ADP-Glo™ luminescence detection protocol according to the manufacturer’s instructions. Luminescence intensity was recorded using a microplate reader, and inhibition percentages were calculated relative to untreated controls. Concentration-response studies were conducted within the range of 0.1–100 µM [60,61,62].

### 2.3. In Silico Studies

The crystal structure of ABL TK (PDB ID: 2HYY) [64] was obtained from the Protein Data Bank. Protein preparation was completed through the Maestro platform [65], including the addition of missing atoms and assignment of protonation states corresponding to physiological pH. Energy refinement was performed using the OPLS force field. Structures of **F-4** and imatinib were generated, geometry-optimized, and prepared under physiological conditions prior to docking. Molecular docking experiments were carried out with the Glide program in standard precision (SP) mode, and the resulting binding conformations were analyzed to characterize interactions within the ATP-binding pocket of ABL TK [60,61,62]. In addition, pharmacokinetic and toxicity characteristics of **F-4** were predicted using ADMETlab 3.0 [66] and SwissADME [67].

## 3. Results

**Scheme 1:**
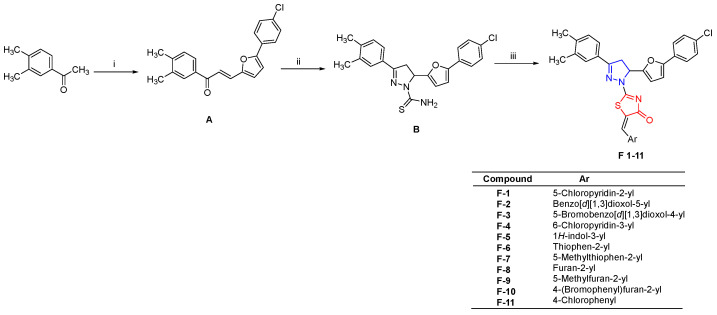
Synthesis of novel thiazolone derivatives (**F1-11**). (i) 5-(4-Chlorophenyl)furan-2-carbaldehyde, NaOH, ethanol, rt, 24 h; (ii) Thiosemicarbazide, NaOH, ethanol, reflux, 8-12 h; (iii) Ar-CHO, ClCH_2_COOH, AcONa, AcOH, reflux, 5 h.

All compounds investigated in this study were newly synthesized. Compound **A** was obtained via *Claisen-Schmidt* condensation of 1-(3,4-dimethylphenyl)ethan-1-one with 5-(4-chlorophenyl)furan-2-carbaldehyde under basic conditions. Subsequently, compound **A** was reacted with thiosemicarbazide in the presence of NaOH to afford compound **B**. Finally, the target thiazolone derivatives (**F1-11**) were synthesized through cyclization with the appropriate aldehydes in the presence of chloroacetic acid, anhydrous sodium acetate, and glacial acetic acid under reflux conditions (Scheme 1). The selection of aldehyde components for the synthesis of **F1-11** derivatives was designed to explore the structure–activity relationship (SAR) by introducing diverse aromatic and heteroaromatic substituents. Both electron-donating and electron-withdrawing groups were incorporated to evaluate their influence on the biological activity and physicochemical properties of the target compounds. In addition, heterocyclic moieties such as furan, thiophene, pyridine, indole, and benzodioxole were included to investigate their potential contribution to binding interactions within the ABL TK active site. This structural diversity was aimed at providing a broad chemical space to identify key substituent effects on anticancer activity.

**Figure 3:**
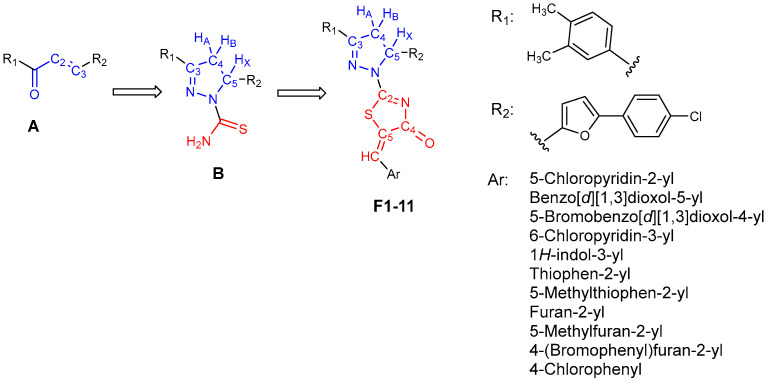
NMR diagram for compounds **A**, **B**, and **F1-11**.

The structures of compounds **A**, **B**, and **F1-11** were confirmed by spectroscopic analyses, including (^1^H nuclear magnetic resonance, NMR), ^13^C NMR, and high-resolution mass spectra (HRMS). In the ^1^H NMR spectrum of compound **A**, the characteristic propenone protons appeared as two doublets at 7.49 ppm (*J* = 15.5 Hz) and 7.58 ppm (*J* = 15.2 Hz), consistent with a trans-α,β-unsaturated carbonyl system, along with the expected aromatic proton signals. In the ^13^C NMR spectrum, the C_2_, C_3_, and carbonyl carbons of the propenone moiety were observed at 119.6 ppm, 126.2 ppm, and 189.6 ppm, respectively (Figure 3). For compound **B**, the presence of the pyrazoline ring was confirmed by three characteristic proton signals. The H_A_, H_B_, and H_X_ protons appeared as doublets of doublets at 3.51 ppm (*J_AB_* = 17.9 Hz, *J_AX_* = 3.3 Hz), 3.69 (1H, dd, *J_BA_* = 17.7 Hz, *J_BX_* = 11.5 Hz), and 6.16 ppm (*J_AX_* = 3.6 Hz, *J_BX_* = 11.3Hz), respectively. These signals are indicative of the typical ABX spin system of the pyrazoline ring. The corresponding ^13^C NMR signals for the C_3_, C_4_, and C_5_ carbons were observed at 156.6 ppm, 39.9 ppm, and 56.9 ppm, respectively. Additionally, the thiocarbonyl (C=S) carbon resonance at 176.4 ppm further supported the proposed structure. The absence of observable amine proton signals may be attributed to rapid exchange or solvent effects. The formation of the thiazolone derivatives (**F1-11**) from compound **B** was confirmed by the disappearance of the thiocarbonyl signal and the appearance of new characteristic signals. The carbonyl carbons (C=O) of the thiazolone ring appeared in the range of 179.9–188.3 ppm, while the imine (C=N) and olefinic (C=CH) carbons were detected at 171.6–181.7 ppm and 151.7–155.5 ppm, respectively. Furthermore, the persistence of the pyrazoline ABX proton system and the corresponding carbon signals (C_3_, C_4_, and C_5_), together with the expected aromatic proton and carbon resonances associated with the newly introduced substituents, confirmed the successful formation of the **F1-11** derivatives (Figure 3).

**Figure 4:**
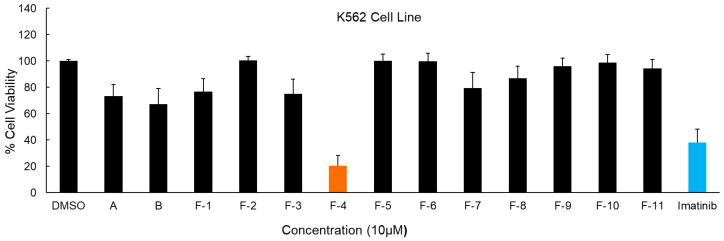
Cytotoxic effects of synthesized compounds (**A**, **B**, and **F1-11**) on the K562 cell line at 10 µM. Cell viability (%) was determined following treatment with the indicated compounds and compared to the DMSO control and imatinib. Data are presented as mean ± SD.

The antiproliferative effects of all synthesized compounds (**A**, **B**, and **F1-11**) were evaluated against the K562 cell line at a concentration of 10 µM. As shown in Figure 4, the DMSO control maintained approximately 100% cell viability, confirming the stability of the assay conditions. The intermediate compounds **A** and **B** exhibited moderate cytotoxic effects, reducing cell viability to around 72–75% and 65–68%, respectively, indicating that partial activity is retained at the precursor level. Among the tested derivatives, **F-4** demonstrated the most pronounced antiproliferative effect, reducing cell viability to nearly 20%, which clearly indicates strong cytotoxic potency. Compounds **F-1** and **F-3** showed moderate activity, maintaining cell viability in the range of approximately 75–78%. Similarly, **F-7** and **F-8** exhibited modest effects, with viability values around 80–86%. In contrast, compounds **F-2**, **F-5**, **F-6**, **F-9**, **F-10**, and **F-11** showed minimal or negligible cytotoxicity, as their viability values remained close to the control group (approximately 95–100%). Importantly, the reference drug imatinib reduced cell viability to approximately 38%, indicating that **F-4** exhibited substantially stronger antiproliferative activity under the same experimental conditions.

**Figure 5:**
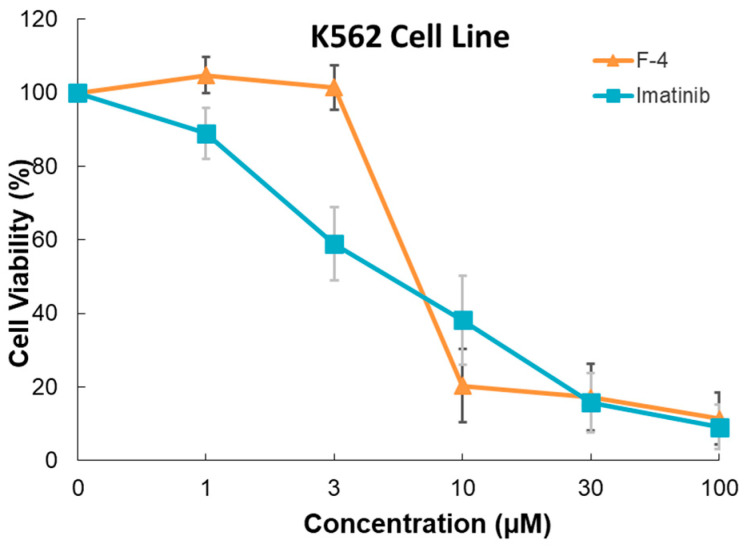
Dose–response effects of **F-4** on K562 CML cells. Cells were treated with increasing concentrations of **F-4** (1–100 µM), and cell viability (%) was determined and compared with imatinib. Data are presented as mean ± SD.

Prompted by its marked cytotoxic activity at 10 µM, **F-4** was subjected to a comprehensive dose–response evaluation across an extended concentration range in K562 CML cells, using imatinib as a comparator. As shown in Figure 5, **F-4** exhibited a clear dose-dependent inhibitory effect on cell viability. At lower concentrations (1–3 µM), **F-4** displayed minimal cytotoxicity, with cell viability remaining close to control levels (~100%). However, a sharp decline in viability was observed at 10 µM, where **F-4** reduced cell survival to approximately 20%, indicating a strong antiproliferative effect. Further increases in concentration (30–100 µM) resulted in sustained suppression of cell viability, reaching approximately 15% and 10%, respectively. In comparison, imatinib demonstrated a more gradual dose-dependent response, with cell viability decreasing to ~88% at 1 µM, ~58% at 3 µM, and ~38% at 10 µM, followed by further reductions at higher concentrations. Notably, **F-4** exhibited markedly greater cytotoxic potency than imatinib at 10 µM, suggesting a steeper dose–response profile and enhanced efficacy at this concentration. Overall, these findings indicate that **F-4** is a highly potent inhibitor of K562 cell proliferation, with a strong and rapid cytotoxic effect emerging at mid-range concentrations.

The IC_50_ values provided a quantitative confirmation of the antiproliferative activity of **F-4** against K562 cells. **F-4** exhibited an IC_50_ value of 6.85 ± 1.36 µM, while imatinib showed an IC_50_ value of 5.20 ± 1.48 µM (Table 1). Although imatinib displayed slightly higher potency, as expected for an established therapeutic agent, the IC_50_ value of **F-4** was relatively close to that of imatinib. This finding suggests that **F-4** possesses notable growth-inhibitory activity against K562 CML cells.

**Figure 6:**
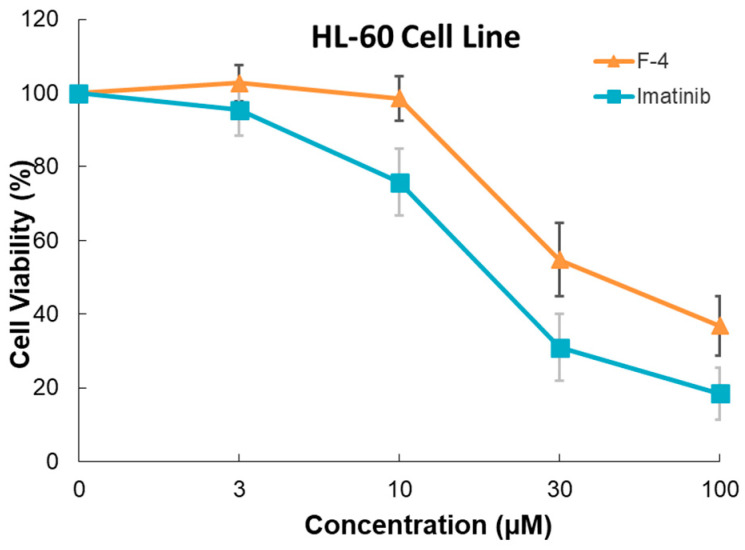
Dose–response effects of **F-4** on HL-60 AML cells. Cells were treated with increasing concentrations of **F-4** (3–100 µM), and cell viability (%) was determined and compared with imatinib. Data are presented as mean ± SD.

To explore the potential selectivity of **F-4** toward CML cells, its antiproliferative activity was further assessed in HL-60 AML cells in comparison with imatinib. As shown in Figure 6, **F-4** exhibited a dose-dependent reduction in cell viability; however, its overall cytotoxic effect was less pronounced than that observed in K562 cells. At lower concentrations (3–10 µM), **F-4** showed minimal activity, with cell viability remaining close to control levels. A more evident decline was observed at higher concentrations, with viability decreasing to approximately 55% at 30 µM and 35% at 100 µM. In contrast, imatinib demonstrated a stronger and more consistent inhibitory effect across the tested concentration range, reducing cell viability to ~75% at 10 µM, ~30% at 30 µM, and ~18% at 100 µM. Notably, at equivalent concentrations, **F-4** exhibited weaker cytotoxicity in HL-60 cells compared to K562 cells, suggesting a degree of preferential activity toward CML cells.

The IC_50_ values obtained in HL-60 cells further supported the observed dose–response trends. **F-4** exhibited an IC_50_ value of 33.44 ± 3.84 µM, whereas imatinib showed a substantially lower IC_50_ value of 17.85 ± 2.55 µM, indicating higher potency of the reference drug in this cell line (Table 1). The markedly higher IC_50_ value of **F-4** compared to that observed in K562 cells suggests reduced sensitivity of HL-60 cells to this compound.

**Figure 7:**
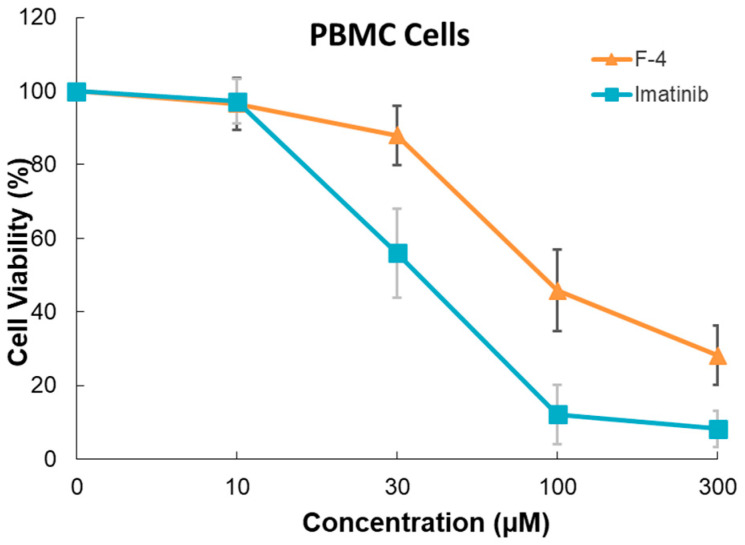
Cytotoxic effects of **F-4** on healthy PBMCs. Cells were treated with increasing concentrations of **F-4** (10–300 µM), and cell viability (%) was determined and compared with imatinib. Data are presented as mean ± SD.

To gain insight into its selectivity toward cancerous cells, the cytotoxic effects of **F-4** were further examined in healthy peripheral blood mononuclear cells (PBMCs) and compared with those observed in K562 cells. **F-4** displayed a concentration-dependent reduction in PBMC viability; however, its cytotoxic impact on healthy cells was notably less pronounced at lower and mid-range concentrations. At 10 µM, PBMC viability remained high (~95%), indicating minimal toxicity under conditions where significant antiproliferative activity was observed in K562 cells. A gradual decline in viability was evident at higher concentrations, with PBMC viability decreasing to approximately 88% at 30 µM, 45% at 100 µM, and 28% at 300 µM. In contrast, imatinib demonstrated a more substantial reduction in PBMC viability across the same concentration range, particularly at higher doses, where viability dropped to nearly 10% at 100 µM and below 10% at 300 µM (Figure 7).

The effects of the compounds on healthy PBMCs were further characterized by calculating IC_50_ and SI values. **F-4** displayed an IC_50_ of 88.77 ± 10.08 µM, while imatinib showed a lower IC_50_ value of 32.92 ± 4.12 µM, indicating a stronger cytotoxic impact of the reference drug on normal cells (Table 1). In line with this trend, the SI values were determined to be 13.0 for **F-4** and 6.0 for imatinib (Table 1). This difference highlights a wider margin between the antiproliferative effect of **F-4** on cancer cells and its impact on healthy PBMCs.

**Figure 8:**
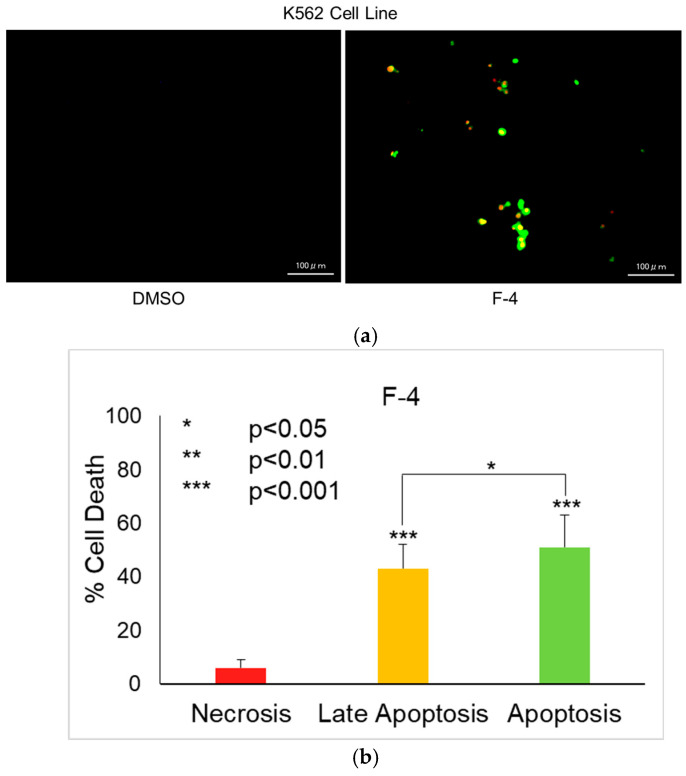
Effects of **F-4** on cell death pathways in K562 cells after 12 h of treatment. (**a**) Fluorescence microscopy images demonstrating the distribution of apoptotic and necrotic cell populations, where viable/apoptotic progression is distinguished by green (early apoptosis), yellow (late apoptosis), and red (necrosis) signals. (**b**) Bar graph summarizing the proportion of cells undergoing early apoptosis, late apoptosis, and necrosis. Values are given as mean ± SD, and statistical differences relative to the untreated control are indicated (* *p* < 0.05; ** *p* < 0.01; *** *p* < 0.001).

The mode of cell death Induced by **F-4** in K562 cells was investigated using fluorescence-based Annexin V/ethidium homodimer staining. According to this assay, apoptotic, necrotic, and late apoptotic/necrotic cells were identified by green, red, and yellow fluorescence signals, respectively (Figure 8a). Quantitative analysis showed that 51% of the cell population was in early apoptosis, while 43% progressed to late apoptosis, whereas necrotic cells accounted for only 6% of the total population. Both early and late apoptotic populations were significantly higher than necrotic cells. In addition, a statistically significant difference was observed between early and late apoptosis (* *p* < 0.05) (Figure 8b). These results indicate that **F-4** predominantly induces apoptotic cell death, with a substantial proportion of cells progressing from early to late apoptosis.

**Figure 9:**
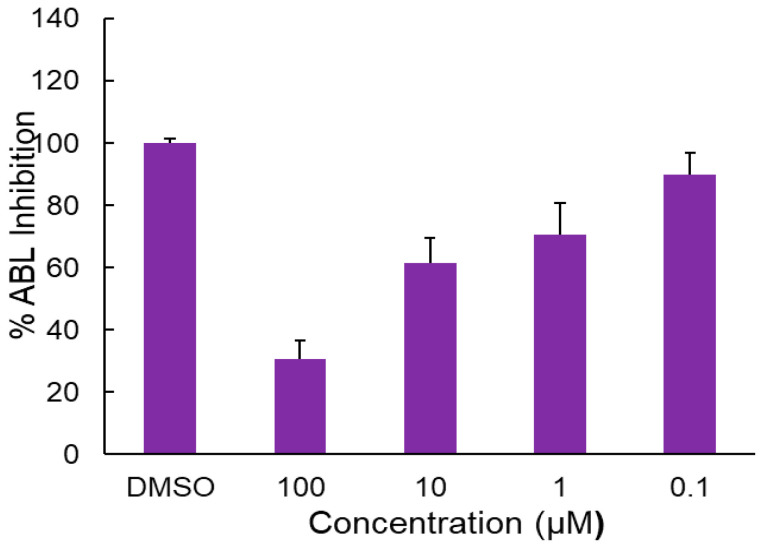
Concentration-dependent inhibition of ABL TK by **F-4**.

The effect of **F-4** on ABL TK activity was evaluated to explore whether its antiproliferative activity in K562 cells may be associated with ABL TK inhibition. When the data were expressed as percentage inhibition, **F-4** showed a concentration-dependent inhibitory effect on ABL TK activity. **F-4** produced limited inhibition at the lowest tested concentration, with approximately 10% inhibition at 0.1 µM. The inhibitory effect increased to about 30% at 1 µM and reached approximately 39% at 10 µM. The strongest effect was observed at 100 µM, where F-4 inhibited ABL TK activity by nearly 70%. These results indicate that **F-4** is able to suppress ABL TK activity, particularly at higher concentrations. This kinase inhibitory activity may contribute, at least in part, to the cytotoxic effect observed in K562 CML cells (Figure 9).

**Figure 10:**
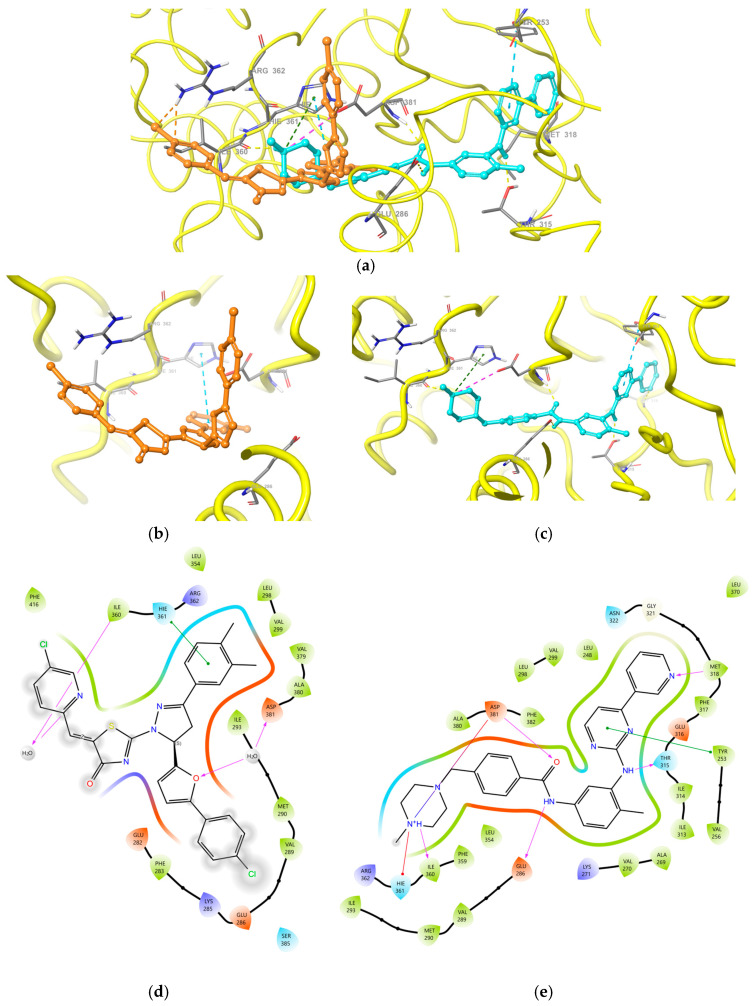
(**a**) Three-dimensional binding mode of **F-4** and imatinib (coloured in orange and turquoise, respectively) within the ABL TK active site. (**b**) Three-dimensional interaction map of **F-4**. (**c**) Three-dimensional interaction profile of imatinib. (**d**) Two-dimensional interaction map of **F-4**. (**e**) Two-dimensional interaction profile of imatinib (Purple lines represent hydrogen bonds; green lines represent π–π interactions).

Molecular docking was carried out to clarify the possible binding pattern of **F-4** within the ATP-binding pocket of ABL TK (PDB ID: 2HYY) [64], and its binding orientation was compared with that of imatinib. The docking model showed that **F-4** was well accommodated within the catalytic pocket of ABL TK, adopting an extended conformation along the binding cleft. The compound was surrounded by several key residues, including Ile293, Leu298, Val299, Ile360, His361, Arg362, Phe382, and Asp381, indicating that hydrophobic and polar contacts contribute to its stabilization within the binding site (Figure 10a–c). In the 2D interaction map, **F-4** (Figure 10d) formed interactions with His361 and Asp381 through polar contact networks, while its aromatic moieties were positioned close to hydrophobic residues such as Ile293, Leu298, Val299, Ile360, Val379, Ala380, and Phe382. These contacts suggest that **F-4** can occupy the ATP-binding region and engage the surrounding hydrophobic pocket. However, compared with imatinib, **F-4** displayed a more limited interaction pattern and did not reproduce some of the classical binding contacts observed for the reference inhibitor. The docking pose of imatinib (Figure 10e) showed a more extensive interaction network within the same pocket. Imatinib interacted with residues such as Met318, Thr315, Tyr253, Glu286, Asp381, and His361, which are known to contribute to stable binding in the ABL TK domain. The 3D overlay further demonstrated that both ligands occupy overlapping regions of the ATP-binding site, although imatinib extends more deeply into additional subpockets and establishes a broader set of stabilizing interactions. The docking score of **F-4** was calculated as −7.179 kcal/mol, whereas imatinib exhibited a significantly more favorable docking score of −12.268 kcal/mol, indicating the comparatively weaker predicted binding affinity of **F-4** toward the ABL TK active site. Overall, the docking results suggest that **F-4** can fit into the ABL TK active site and form relevant contacts with residues lining the ATP-binding pocket. Although its interaction network appears less extensive than that of imatinib, the observed binding mode is consistent with the experimental ABL TK inhibitory activity of **F-4**.

## 4. Discussion

CML remains a well-established model for targeted therapy; however, despite the success of ABL TKIs, key limitations persist. Resistance development, survival of leukemic stem cell populations, and long-term tolerability issues continue to limit durable treatment responses. These considerations underscore the importance of identifying structurally novel molecules that may act through complementary or partially distinct mechanisms while preserving an acceptable therapeutic balance.

Previous investigations of thiazolone-based derivatives have predominantly focused on breast and colon cancer models; however, hybrid structures incorporating pyrazoline units have consistently demonstrated a broader anticancer profile. In this context, Havrylyuk et al. (2009) [50] reported that compound 6 exhibited selective activity toward leukemia cell lines, suggesting that hydroxyphenyl substitution on both the pyrazoline and thiazolone scaffolds may contribute to lineage-specific effects. In a subsequent study, Havrylyuk et al. (2012) [51] described a series of pyrazoline-thiazolone hybrids evaluated by the National Cancer Institute (NCI), where several compounds displayed broad-spectrum cytotoxicity across multiple cancer types, including leukemia, lung, colon, CNS, ovarian, and renal cell lines. Among these, compound 10 emerged as the most potent derivative, demonstrating strong antiproliferative activity at submicromolar levels. Similarly, Khalil et al. (2013) [52] identified compound 3i as a highly active derivative, emphasizing the contribution of methoxyphenyl substitution to enhanced biological response. In another study, Avdieiev et al. (2014) [53] evaluated thiazolidinone derivatives against mantle cell lymphoma (MCL) cells and reported that compound ID 4527 exhibited notable antitumor activity with a low micromolar IC_50_ value. In addition, Meleddu et al. (2018) [54] investigated isatin-dihydropyrazole thiazolone derivatives and identified EMAC4001 as the most active compound, showing potent effects across multiple cancer cell lines along with acceptable activity toward normal fibroblasts.

Notably, literature examples such as ID 4527 highlight the potential importance of halogen substitution on both pyrazoline and thiazolone frameworks. This observation is particularly relevant to the present study, as **F-4** also incorporates a halogenated heteroaromatic substituent, suggesting that such structural features may contribute to the observed antiproliferative activity.

In the current work, the applied synthetic strategy enabled the successful assembly of the pyrazoline-thiazolone framework while preserving key structural motifs throughout the reaction sequence. The transformation pathway, involving stepwise formation of the pyrazoline intermediate followed by cyclization into the thiazolone core, supports the structural integrity of the final compounds. The deliberate incorporation of electronically diverse aromatic and heteroaromatic substituents further expanded the chemical space, allowing systematic evaluation of substituent-driven effects on biological activity.

Consistent with this design approach, the biological response observed across the series was strongly dependent on structural variation. Among the synthesized compounds, **F-4** displayed a clearly differentiated activity profile, suggesting that its molecular architecture provides a more favorable alignment of electronic distribution and steric compatibility.

Structure–activity analysis (SAR) analysis suggests that both electronic and steric properties of the substituents contribute significantly to the observed biological activity profile. SAR indicates that heteroaromatic substituents, particularly the 6-chloropyridin-3-yl moiety, may enhance interaction potential with intracellular targets. The moderate responses observed for **F-1** and **F-3** further support the contribution of related groups, such as 5-chloropyridin-2-yl and 5-bromobenzo[d][1,3]dioxol-4-yl substitutions, respectively. However, the lack of comparable activity in other heteroatom-containing derivatives indicates that the presence of such functionalities alone is not sufficient to ensure high potency. Instead, the observed activity appears to arise from a coordinated interplay between electronic characteristics, substituent orientation, and overall molecular conformation. This suggests that the contribution of chloropyridinyl groups is context-dependent within the structural framework. Moreover, the structural transformation from intermediates **A** and **B** to the final thiazolone derivative **F-4** is associated with a marked enhancement in biological activity, highlighting the importance of thiocarbamoyl cyclization into the thiazolone ring for anti-CML activity.

The response behavior of **F-4** further distinguishes it from the remaining compounds. Rather than exhibiting a gradual effect, its activity appears to intensify beyond a certain threshold, which may reflect differences in intracellular accumulation or engagement with specific molecular pathways. This pattern suggests that **F-4** may activate key cellular processes once sufficient intracellular concentration is achieved.

Differences observed between cell lines provide additional insight into their biological profiles. The reduced response in HL-60 cells compared to K562 cells suggests a degree of selectivity, potentially linked to ABL TK-driven signaling pathways. In parallel, the relatively limited effect on PBMCs indicates that the observed cytotoxicity is not predominantly associated with nonspecific cellular damage, supporting a more selective mode of action.

Mechanistically, the predominance of apoptotic features suggests that **F-4** primarily induces regulated cell death rather than direct cytotoxic disruption. This behavior is generally associated with more controlled anticancer effects and is consistent with the observed selectivity pattern.

Evaluation of ABL TK activity provides further mechanistic context. Although **F-4** exhibited measurable inhibitory activity, its effect appears less extensive than that of established inhibitors, indicating that kinase targeting may contribute only partially to its overall biological effect. In particular, the pronounced antiproliferative activity observed at 10 µM relative to the moderate ABL TK inhibition suggests the possible involvement of additional intracellular mechanisms. Previous studies have demonstrated that, besides direct BCR-ABL inhibition, imatinib can also modulate downstream signaling pathways associated with cell survival and apoptosis, including PI3K/AKT, RAS/MAPK, and JAK/STAT pathways, ultimately promoting apoptotic cell death. Similarly, thiazolone- and pyrazoline-based derivatives have been reported to exhibit multi-target anticancer activities involving apoptosis-related signaling and cell cycle regulation. Therefore, the strong apoptotic response induced by **F-4** suggests that its antiproliferative activity may result from both partial ABL TK inhibition and additional intracellular signaling effects. However, further mechanistic investigations are required to clarify the precise pathways involved.

Molecular docking analysis aligns with these findings, demonstrating that **F-4** can be accommodated within the ATP-binding region of ABL TK while forming interactions with residues in the catalytic pocket. However, compared to imatinib, its interaction network appears less extensive, lacking several stabilizing contacts associated with high-affinity binding. This structural limitation may explain the moderate kinase inhibition and suggests that further optimization of substituent orientation could improve binding efficiency.

Preliminary in silico ADMET evaluation of **F-4** was performed using SwissADME and ADMETlab platforms. The analyses suggested several favorable properties, including acceptable molecular flexibility, absence of predicted P-gp substrate liability, and suitable physicochemical parameters supporting its potential as a lead structure. However, the compound also exhibited high lipophilicity and poor predicted aqueous solubility, indicating that further structural optimization will be required to improve its pharmacokinetic and drug-like properties. Therefore, **F-4** should be considered a promising preliminary lead compound rather than a fully optimized drug candidate.

Taken together, these findings position **F-4** as a promising lead compound within this thiazolone-based series. Its distinct activity profile, context-dependent selectivity, apoptosis-associated mechanism, and measurable interaction with ABL TK collectively support its potential for further development. At the same time, the current data indicate that structural refinement will be necessary to enhance target engagement and optimize pharmacological performance.

## 5. Conclusions

In this study, a series of novel thiazolone-based derivatives was designed and synthesized to evaluate their antiproliferative potential against CML cells. Among the investigated compounds, **F-4** stood out as the most promising candidate, displaying a distinct activity profile within the series. The findings indicate that structural features, particularly the nature and positioning of heteroaromatic substituents, play a key role in shaping anticancer activity. The results suggest that **F-4** offers a favorable balance between antiproliferative efficacy and selectivity, with a response pattern that differs from conventional inhibitors. Mechanistic observations point to apoptosis induction as a major contributor to its biological effect, while ABL TK inhibition may play a supportive role, as also indicated by docking analysis. At the same time, the interaction profile implies that further structural refinement could enhance target engagement. Overall, this study provides meaningful insight into the structure–activity relationships of thiazolone-based hybrids and highlights **F-4** as a promising lead compound. Future efforts focusing on structural optimization and more detailed mechanistic studies may support the development of improved anti-CML agents.

## Figures and Tables

**Table 1:** IC_50_ values and selectivity indices (SI) of **F-4** and imatinib.

Compound	IC_50_ Values (μM)			SI *
	K562	HL-60	PBMCs	
**F-4**	6.85 ± 1.36	33.44 ± 3.84	88.77 ± 10.08	13.0
**Imatinib**	5.20 ± 1.48	17.85 ± 2.55	32.92 ± 4.12	6.0

* SI = IC_50_ for PBMC/IC_50_ for K562 cell line.

## Data Availability

The original contributions presented in this study are included in the article. Further inquiries can be directed to the corresponding author(s).

## Supplementary Materials

The following supporting information can be downloaded at: https://www.mdpi.com/article/10.3390/pharmaceutics18060709/s1, Figure S1: ^1^H NMR Spectrum of compound **A**; Figure S2: ^13^C NMR Spectrum of compound **A**; Figure S3: Mass Spectrum of compound **A**; Figure S4: ^1^H NMR Spectrum of compound **B**; Figure S5: ^13^C NMR Spectrum of compound **B**; Figure S6: Mass Spectrum of compound **B**; Figure S7: ^1^H NMR Spectrum of **F-1**; Figure S8: ^13^C NMR Spectrum of **F-1**; Figure S9: Mass Spectrum of **F-1**; Figure S10: ^1^H NMR Spectrum of **F-2**; Figure S11: ^13^C NMR Spectrum of **F-2**; Figure S12: Mass Spectrum of **F-2**; Figure S13: ^1^H NMR Spectrum of **F-3**; Figure S14: ^13^C NMR Spectrum of **F-3**; Figure S15: Mass Spectrum of **F-3;** Figure S16: ^1^H NMR Spectrum of **F-4**; Figure S17: ^13^C NMR Spectrum of **F-4;** Figure S18: Mass Spectrum of **F-4;** Figure S19: ^1^H NMR Spectrum of **F-5**; Figure S20: ^13^C NMR Spectrum of **F-5**; Figure S21: Mass Spectrum of **F-5**; Figure S22: ^1^H NMR Spectrum of **F-6**; Figure S23: ^13^C NMR Spectrum of **F-6**; Figure S24: Mass Spectrum of **F-6**; Figure S25: ^1^H NMR Spectrum of **F-7**; Figure S26: ^13^C NMR Spectrum of **F-7**; Figure S27: Mass Spectrum of **F-7**; Figure S28: ^1^H NMR Spectrum of **F-8;** Figure S29: ^13^C NMR Spectrum of **F-8**; Figure S30: Mass Spectrum of **F-8**; Figure S31: ^1^H NMR Spectrum of **F-9**; Figure S32: ^13^C NMR Spectrum of **F-9**; Figure S33: Mass Spectrum of **F-9**; Figure S34: ^1^H NMR Spectrum of F-10; Figure S35: ^13^C NMR Spectrum of **F-10**; Figure S36: Mass Spectrum of **F-10**; Figure S37: ^1^H NMR Spectrum of **F-11**; Figure S38: ^13^C NMR Spectrum of **F-11**; Figure S39: Mass Spectrum of **F-11**.
