# Supplementary material for: Targeting ABL Tyrosine Kinase in Chronic Myeloid Leukemia: Design, Synthesis, Biological Evaluation, and Computational Studies of Novel Thiazolone Derivatives
Source: Pharmaceutics. 2026 Jun 9;18(6):709. doi: 10.3390/pharmaceutics18060709 (PMC13306063; doi:10.3390/pharmaceutics18060709)
Supplement: Supplementary file 1 [file pharmaceutics-18-00709-s001.zip › pharmaceutics-4316441-supplementary.pdf]

Supplementary Information

argeting ABL Tyrosine Kinase in Chronic Myeloid Leukemia: Design, Synthesis, Biological Evaluation, and Computational Studies of Novel Thiazolone Derivatives

Belgin Sever [1, 2,*] and Halilibrahim Ciftci [2,3,*]

1 Department of Pharmaceutical Chemistry, Faculty of Pharmacy, Anadolu University, Eskisehir 26470, Türkiye

2 Department of Molecular Biology and Genetics, Burdur Mehmet Akif Ersoy University, Istiklal Campus, Burdur 15200, Türkiye

3 Medicinal and Biological Chemistry Science Farm Joint Research Laboratory, Faculty of Life Sciences, Kumamoto University, Kumamoto 862-0973, Japan

* Correspondence: belginsever@anadolu.edu.tr (B.S.); hciftci@mehmetakif.edu.tr (H.C.)

Supplementary Figures

Figure S1: ^1H NMR Spectrum of compound **A**
Figure S2: ^{13}C NMR Spectrum of compound **A**
Figure S3: Mass Spectrum of compound **A**
Figure S4: ^1H NMR Spectrum of compound **B**
Figure S5: ^{13}C NMR Spectrum of compound **B**
Figure S6: Mass Spectrum of compound **B**
Figure S7: ^1H NMR Spectrum of **F-1**
Figure S8: ^{13}C NMR Spectrum of **F-1**
Figure S9: Mass Spectrum of **F-1**
Figure S10: ^1H NMR Spectrum of **F-2**
Figure S11: ^{13}C NMR Spectrum of **F-2**
Figure S12: Mass Spectrum of **F-2**
Figure S13: ^1H NMR Spectrum of **F-3**
Figure S14: ^{13}C NMR Spectrum of **F-3**
Figure S15: Mass Spectrum of **F-3**
Figure S16: ^1H NMR Spectrum of **F-4**
Figure S17: ^{13}C NMR Spectrum of **F-4**
Figure S18: Mass Spectrum of **F-4**
Figure S19: ^1H NMR Spectrum of **F-5**
Figure S20: ^{13}C NMR Spectrum of **F-5**
Figure S21: Mass Spectrum of **F-5**
Figure S22: ^1H NMR Spectrum of **F-6**
Figure S23: ^{13}C NMR Spectrum of **F-6**
Figure S24: Mass Spectrum of **F-6**
Figure S25: ^1H NMR Spectrum of **F-7**
Figure S26: ^{13}C NMR Spectrum of **F-7**
Figure S27: Mass Spectrum of **F-7**
Figure S28: ^1H NMR Spectrum of **F-8**
Figure S29: ^{13}C NMR Spectrum of **F-8**
Figure S30: Mass Spectrum of **F-8**
Figure S31: ^1H NMR Spectrum of **F-9**
Figure S32: ^{13}C NMR Spectrum of **F-9**
Figure S33: Mass Spectrum of **F-9**
Figure S34: ^1H NMR Spectrum of **F-10**
Figure S35: ^{13}C NMR Spectrum of **F-10**
Figure S36: Mass Spectrum of **F-10**
Figure S37: ^1H NMR Spectrum of **F-11**
Figure S38: ^{13}C NMR Spectrum of **F-11**
Figure S39: Mass Spectrum of **F-11**

Figure S1. ^{1}H NMR Spectrum of compound **A**

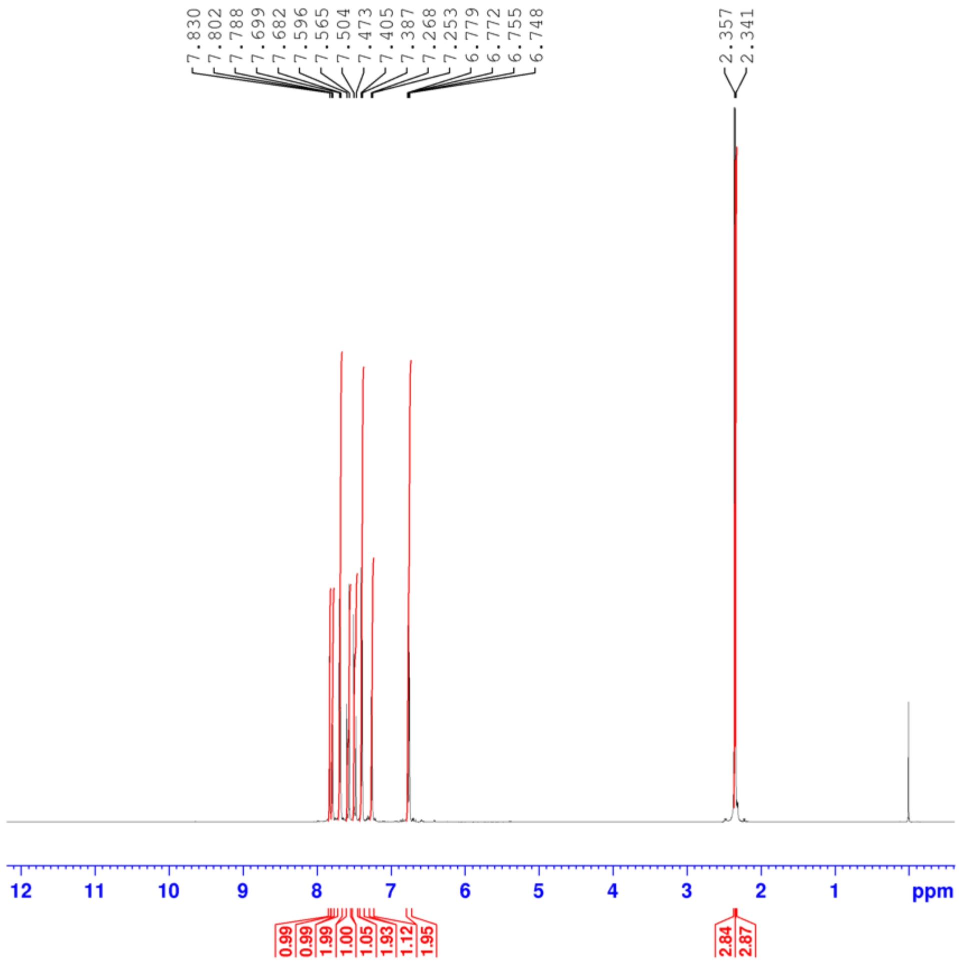

Figure S2. ^{13}C NMR Spectrum of compound **A**

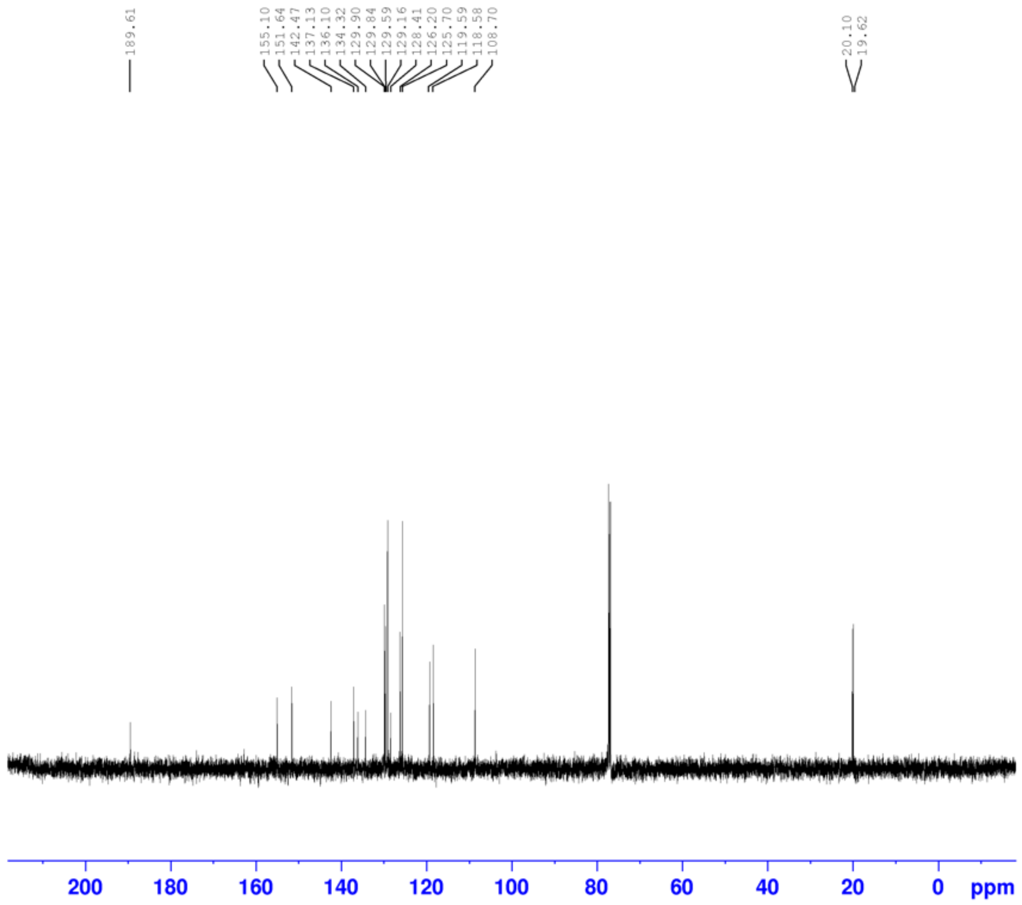

Figure S3: Mass Spectrum of compound **A**

```
Note : CHCl3+NBA
Inlet : Direct                              Ion Mode : FAB+
RT : 1.63 min                               Scan#: (6,9)
Elements : C 200/0, H 200/0, O 3/1, Cl 2/0
Mass Tolerance      : 20ppm, 10mmu if m/z < 500, 20mmu if m/z > 1000
Unsaturation (U.S.) : -0.5 - 100.0

Observed m/z Int%   Err[ppm / mmu]    U.S.  Composition
  336.0923    95.1     +1.9 /  +0.6   13.0  C 21 H 17 O 2 Cl

  337.0989   100.0     -1.9 /  -0.6   12.5  C 21 H 18 O 2 Cl
```

```
[ Theoretical Ion Distribution ]                                    Page: 1
Molecular Formula : C21 H18 O2 Cl
                      (m/z   337.0995, MW   337.8257, U.S. 12.5)
Base Peak :    337.0995, Averaged MW :   337.8267(a),    337.8296(w)

    m/z        INT.
  337.0995 100.0000 ***********************************************************
  338.1029  23.7031 **************
  339.0974  35.0586 ********************
  340.1003   7.8669 *****
  341.1032   1.0059 *
  342.1060   0.0930
  343.1088   0.0067
  344.1116   0.0004
```

Figure S4: ^{1}H NMR Spectrum of compound **B**

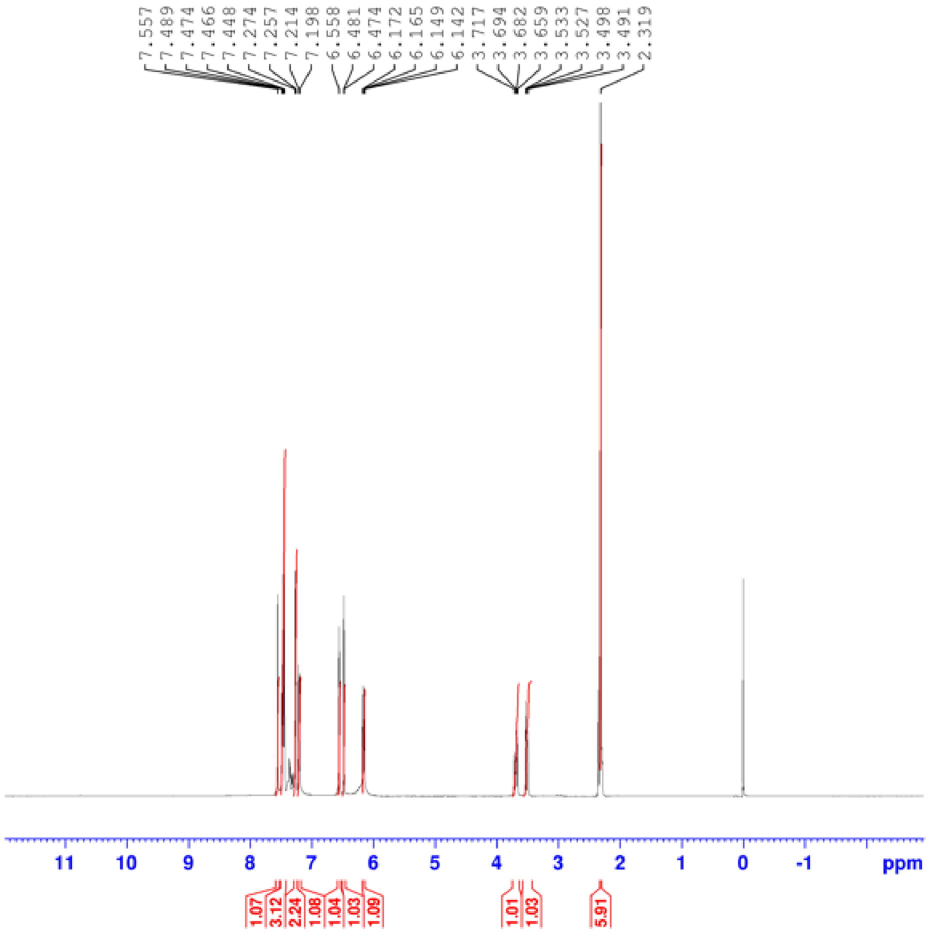

Figure S5: ^{13}C NMR Spectrum of compound **B**

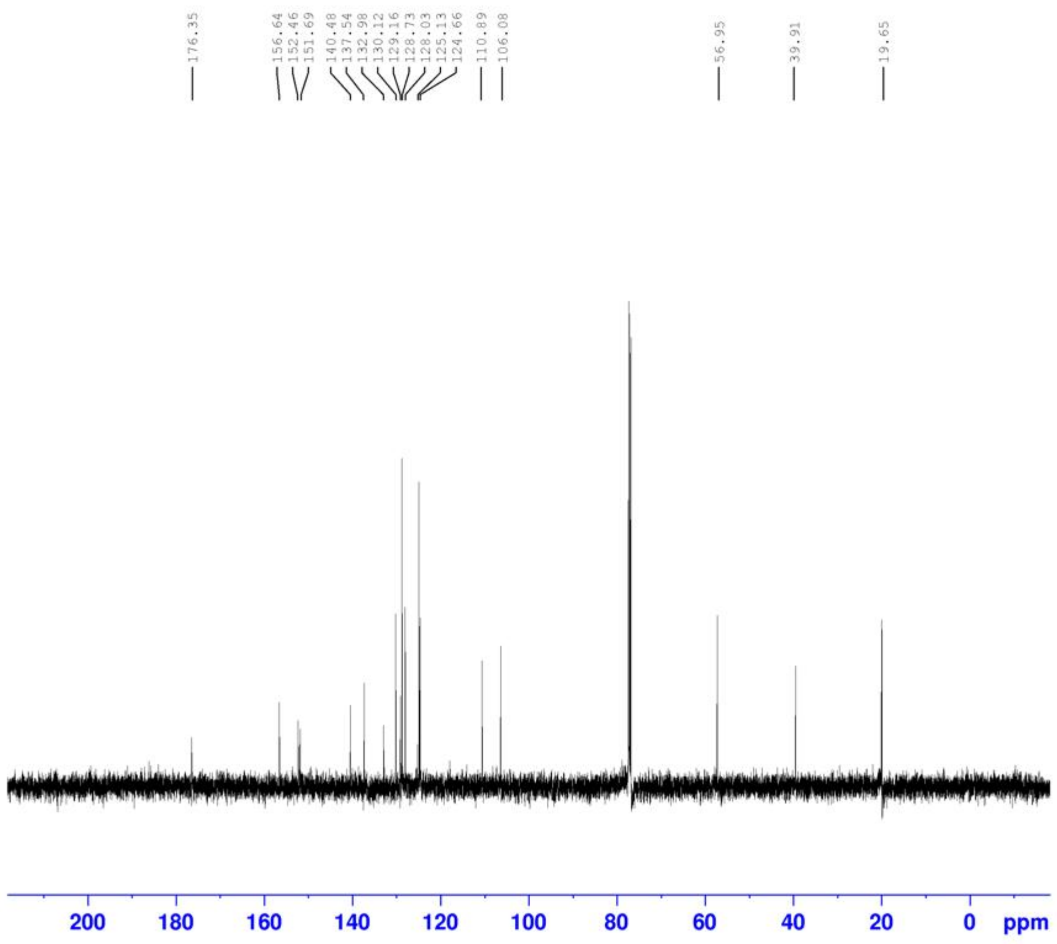

Figure S6: Mass Spectrum of compound **B**

Note : NBA
Inlet : Direct Ion Mode : FAB+
RT : 1.63 min Scan#: (6,9)
Elements : C 200/0, H 200/0, O 2/0, N 4/2, Cl 2/0, S 2/0
Mass Tolerance : 20ppm, 10mmu if m/z < 500, 20mmu if m/z > 1000
Unsaturation (U.S.) : -0.5 - 100.0

Observed m/z	Int%	Err[ppm / mmu]	U.S.	Composition
409.1016	55.4	+9.4 / +3.9	23.5	C 28 H 13 O 2 N 2
		-18.0 / -7.4	23.5	C 27 H 13 O N 4
		-22.5 / -9.2	18.5	C 26 H 18 O N 2 Cl
		+8.2 / +3.4	19.0	C 25 H 16 O N 3 Cl
		-23.7 / -9.7	14.0	C 23 H 21 N 3 Cl 2
		+7.1 / +2.9	14.5	C 22 H 19 N 4 Cl 2
		+1.2 / +0.5	19.5	C 25 H 17 O 2 N 2 S
		+0.0 / +0.0	15.0	C 22 H 20 O N 3 Cl S
		-1.2 / -0.5	10.5	C 19 H 23 N 4 Cl 2 S
		-7.1 / -2.9	15.5	C 22 H 21 O 2 N 2 S 2
		+23.7 / +9.7	16.0	C 21 H 19 O 2 N 3 S 2
		-8.2 / -3.4	11.0	C 19 H 24 O N 3 Cl S 2
		+22.5 / +9.2	11.5	C 18 H 22 O N 4 Cl S 2
		+18.0 / +7.4	6.5	C 17 H 27 O N 2 Cl 2 S 2
		-9.4 / -3.9	6.5	C 16 H 27 N 4 Cl 2 S 2
410.1081	100.0	+6.3 / +2.6	23.0	C 28 H 14 O 2 N 2
		-21.1 / -8.7	23.0	C 27 H 14 O N 4
		+5.1 / +2.1	18.5	C 25 H 17 O N 3 Cl
		+3.9 / +1.6	14.0	C 22 H 20 N 4 Cl 2
		-2.0 / -0.8	19.0	C 25 H 18 O 2 N 2 S
		-3.2 / -1.3	14.5	C 22 H 21 O N 3 Cl S
		+23.0 / +9.5	10.0	C 20 H 24 O N 2 Cl 2 S
		-4.3 / -1.8	10.0	C 19 H 24 N 4 Cl 2 S
		-10.2 / -4.2	15.0	C 22 H 22 O 2 N 2 S 2
		+20.5 / +8.4	15.5	C 21 H 20 O 2 N 3 S 2
		-11.4 / -4.7	10.5	C 19 H 25 O N 3 Cl S 2
		+19.3 / +7.9	11.0	C 18 H 23 O N 4 Cl S 2
		+14.8 / +6.1	6.0	C 17 H 28 O N 2 Cl 2 S 2
		-12.6 / -5.2	6.0	C 16 H 28 N 4 Cl 2 S 2

[Theoretical Ion Distribution] Page: 1
Molecular Formula : C22 H21 O N3 Cl S
(m/z 410.1094, MW 410.9472, U.S. 14.5)
Base Peak : 410.1094, Averaged MW : 410.9469(a), 410.9497(w)

```
   m/z       INT.
410.1094 100.0000 **************************************************
411.1124  26.7136 **************
412.1071  40.0363 *********************
413.1096  10.0257 ******
414.1071   2.7729 **
415.1078   0.4947
416.1089   0.0644
417.1102   0.0066
418.1117   0.0006
```

Figure S7: ^{1}H NMR Spectrum of **F-1**

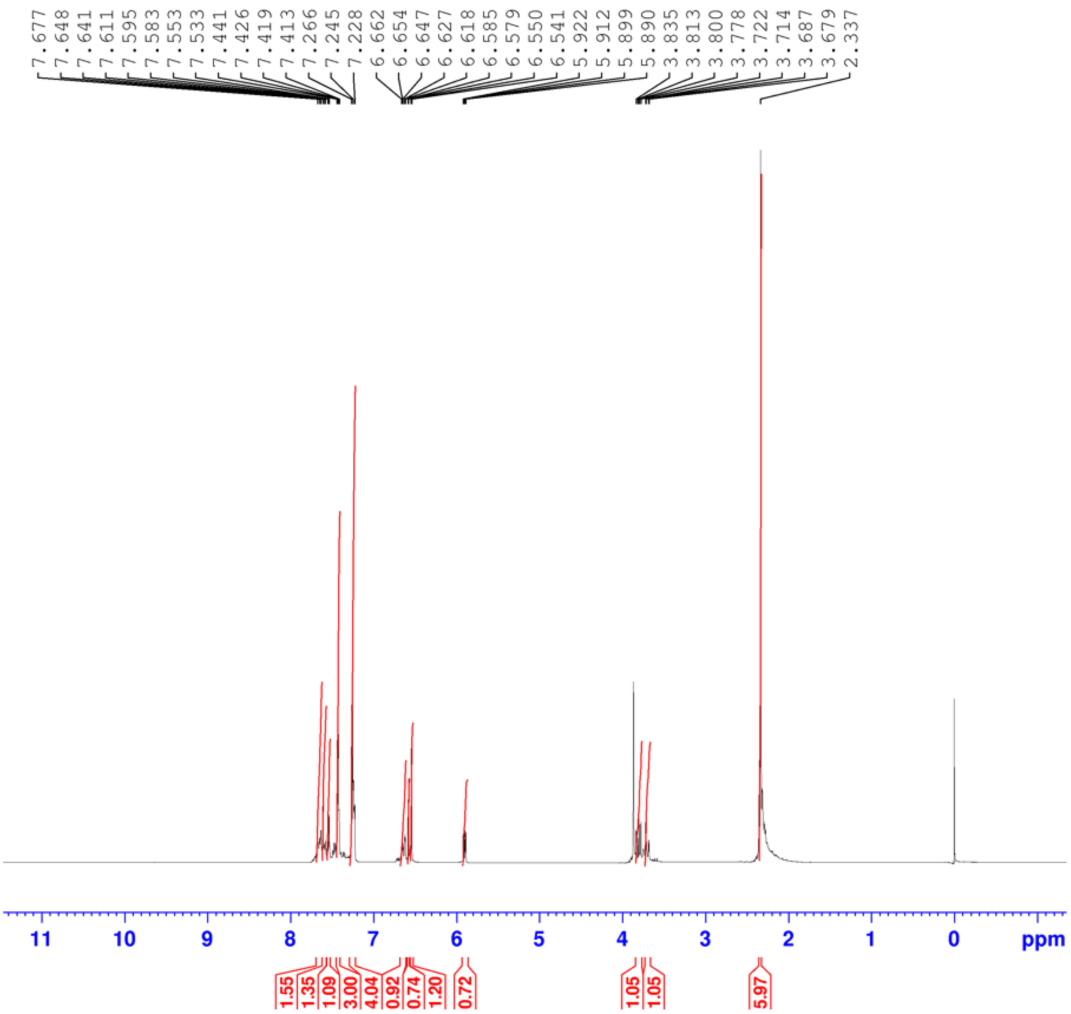

Figure S8: ^{13}C NMR Spectrum of **F-1**

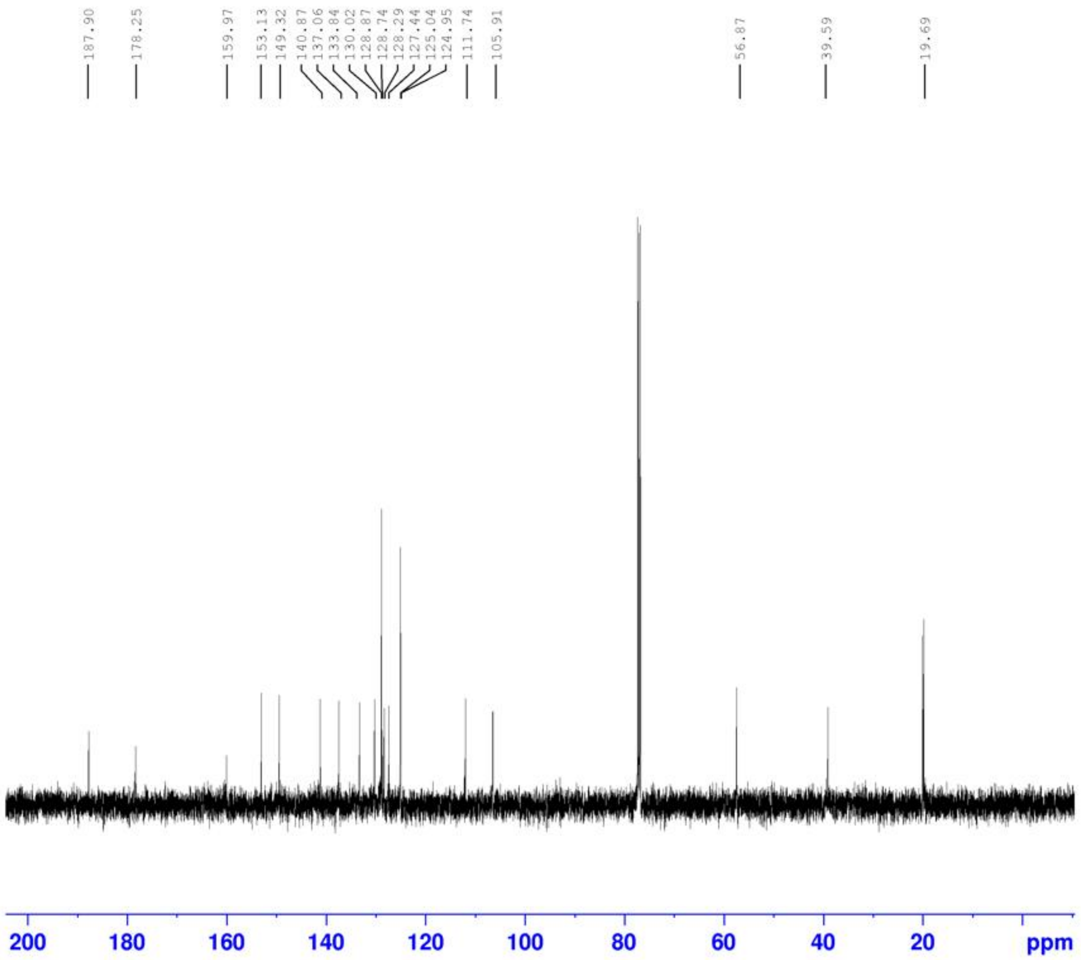

Figure S9: Mass Spectrum of **F-1**

```
Note : NBA
Inlet : Direct                                  Ion Mode : FAB+
RT : 1.00 min                                   Scan#: (3,7)
Elements : C 200/0, H 200/0, O 3/1, N 5/3, Cl 3/1, S 2/0
Mass Tolerance       : 20ppm, 10mmu if m/z < 500, 20mmu if m/z > 1000
Unsaturation (U.S.) : -0.5 - 100.0

Observed m/z  Int%    Err[ppm / mmu]     U.S.  Composition
  573.0901    28.5       +3.7 /  +2.1    30.0  C 36 H 16 O 3 N 3 Cl
                        -15.9 /  -9.1    30.0  C 35 H 16 O 2 N 5 Cl
                        -19.1 / -10.9    25.0  C 34 H 21 O 2 N 3 Cl 2
                         +2.9 /  +1.6    25.5  C 33 H 19 O 2 N 4 Cl 2
                        -19.9 / -11.4    20.5  C 31 H 24 O N 4 Cl 3
                         +2.0 /  +1.2    21.0  C 30 H 22 O N 5 Cl 3
                         -2.2 /  -1.2    26.0  C 33 H 20 O 3 N 3 Cl S
                        +19.8 / +11.3    26.5  C 32 H 18 O 3 N 4 Cl S
                         -3.0 /  -1.7    21.5  C 30 H 23 O 2 N 4 Cl 2 S
                        +18.9 / +10.8    22.0  C 29 H 21 O 2 N 5 Cl 2 S
                        +15.7 /  +9.0    17.0  C 28 H 26 O 2 N 3 Cl 3 S
                         -3.9 /  -2.2    17.0  C 27 H 26 O N 5 Cl 3 S
                         -8.1 /  -4.6    22.0  C 30 H 24 O 3 N 3 Cl S 2
                        +13.9 /  +8.0    22.5  C 29 H 22 O 3 N 4 Cl S 2
                         -8.9 /  -5.1    17.5  C 27 H 27 O 2 N 4 Cl 2 S 2
                        +13.0 /  +7.5    18.0  C 26 H 25 O 2 N 5 Cl 2 S 2
                         +9.8 /  +5.6    13.0  C 25 H 30 O 2 N 3 Cl 3 S 2
                         -9.8 /  -5.6    13.0  C 24 H 30 O N 5 Cl 3 S 2
```

```
[ Theoretical Ion Distribution ]                                  Page: 1
Molecular Formula : C30 H23 O2 N4 Cl2 S
                    (m/z   573.0919, MW   574.5102, U.S. 21.5)
Base Peak :   573.0919, Averaged MW :   574.5099(a),   574.5134(w)

   m/z         INT.
  573.0919 100.0000 ************************************************************
  574.0949  36.0470 *********************
  575.0897  75.0938 *********************************************
  576.0922  25.4740 ***************
  577.0881  17.7392 **********
  578.0898   5.2836 ***
  579.0888   1.3936 *
  580.0891   0.2798
  581.0901   0.0433
  582.0914   0.0054
  583.0930   0.0006
```

Figure S10: ^{1}H NMR Spectrum of **F-2**

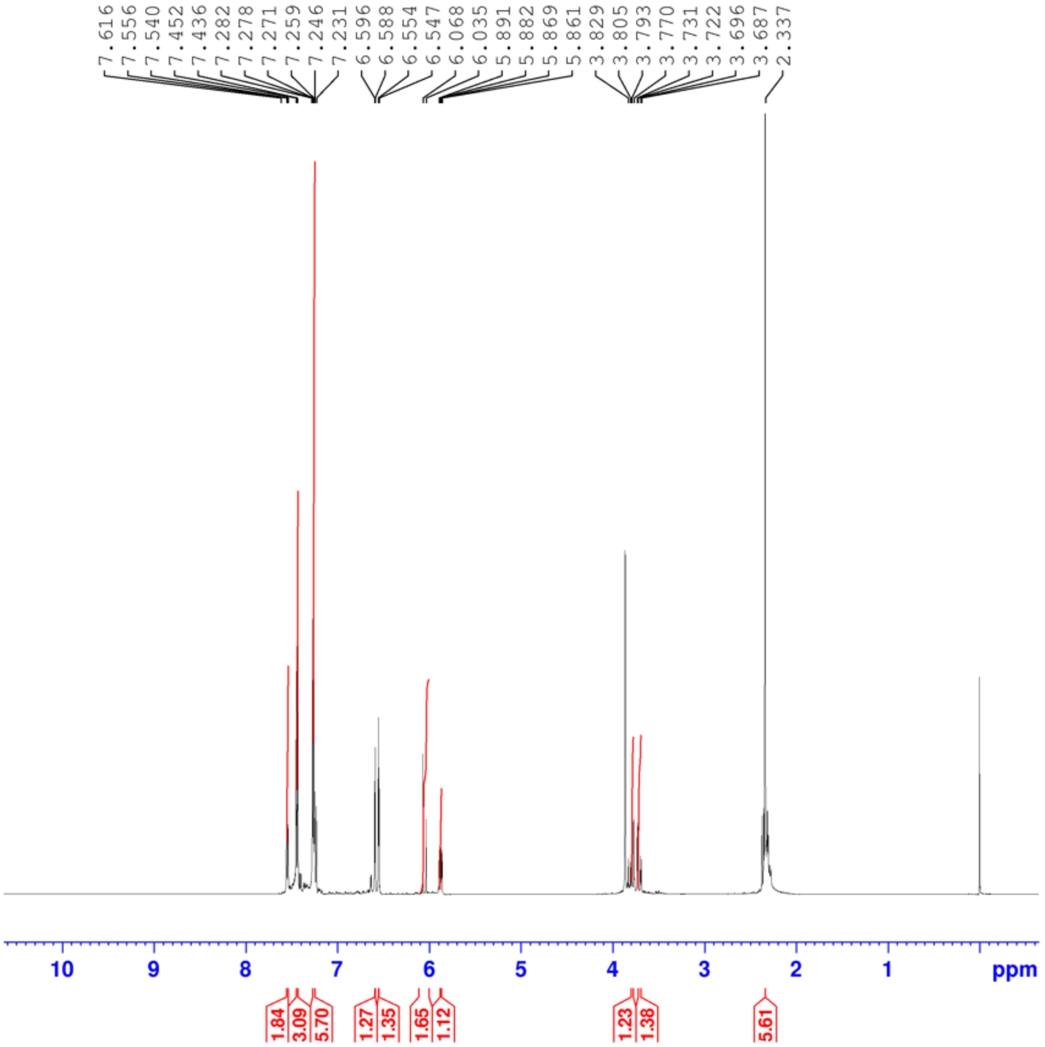

Figure S11: ^{13}C NMR Spectrum of **F-2**

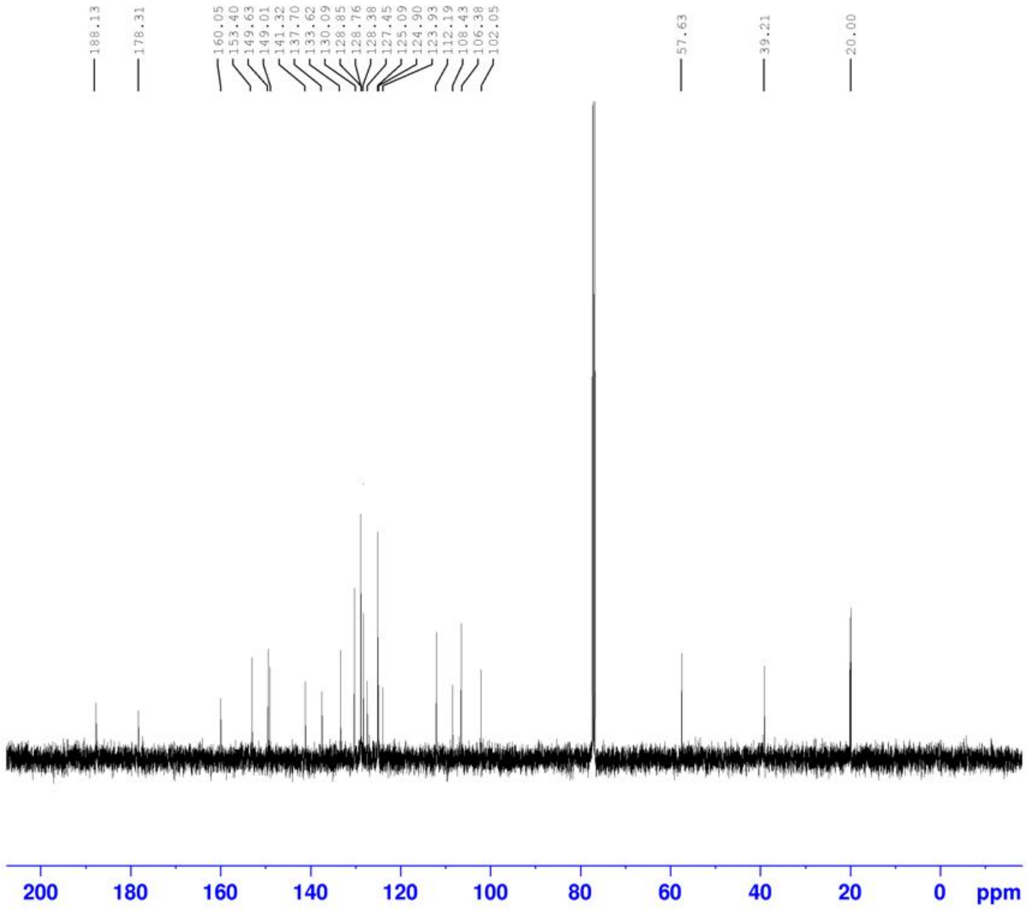

Figure S12: Mass Spectrum of **F-2**

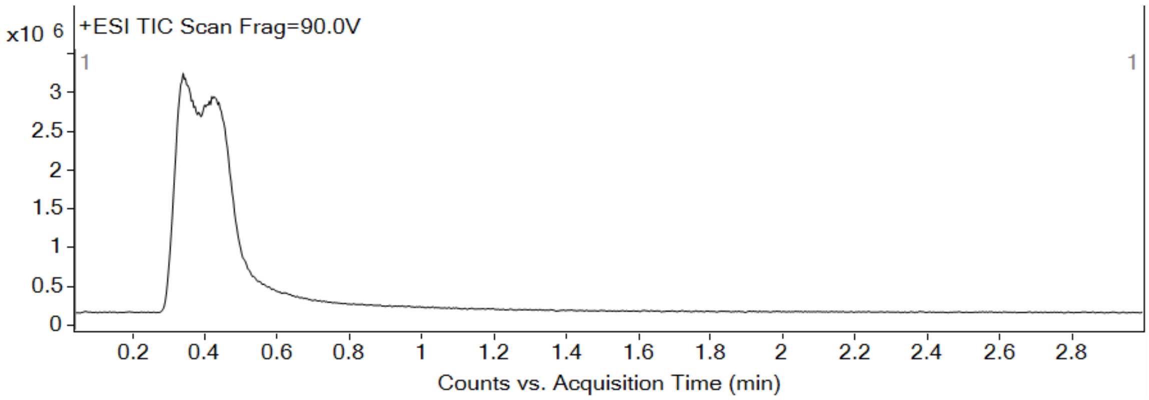

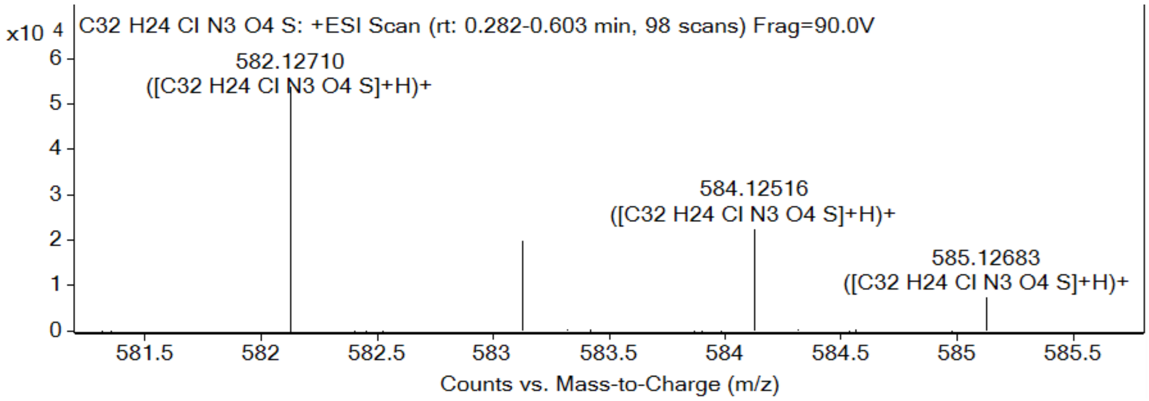

Formula	Best	Mass	Tgt Mass	Diff (ppm)	Ion Species	Score
C32 H24 Cl N3 O4 S	TRUE	581,11962	581,1176	3,47	C32 H25 Cl N3 O4 S	93,05

Figure S13: ^{1}H NMR Spectrum of **F-3**

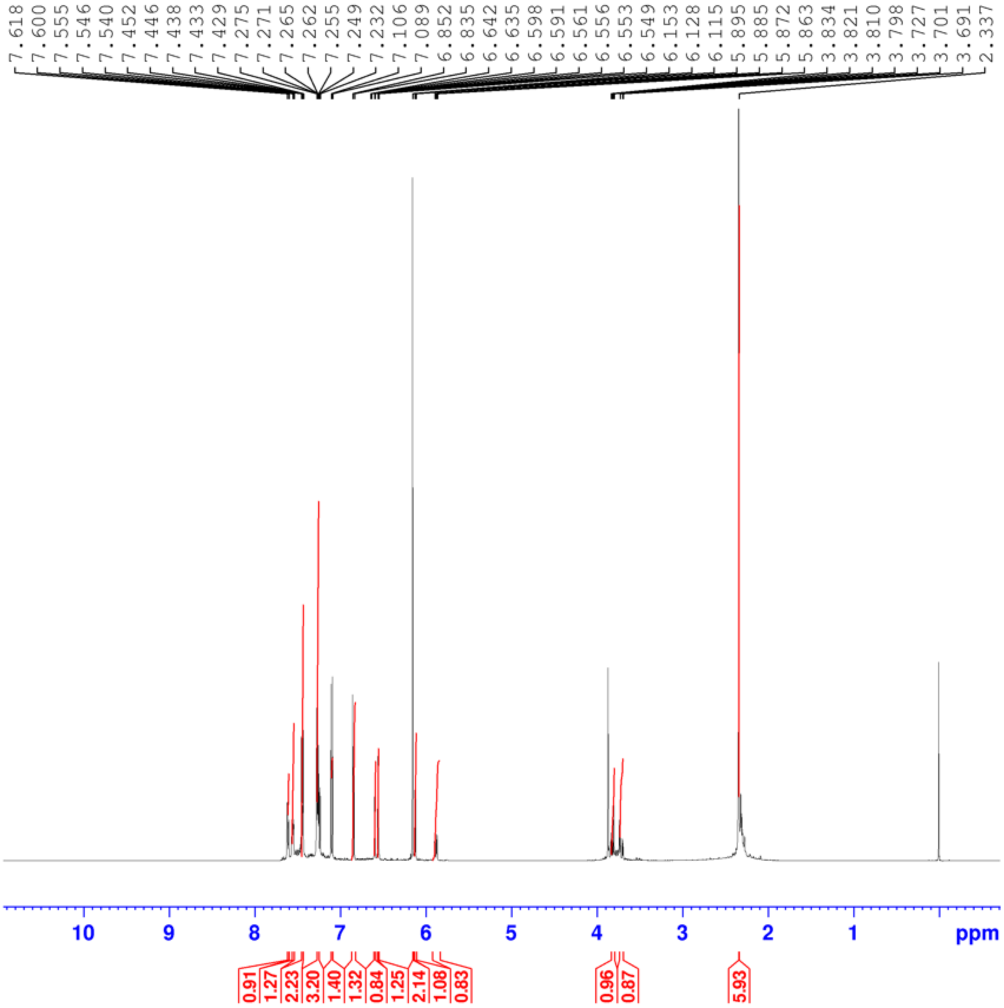

Figure S14: ^{13}C NMR Spectrum of **F-3**

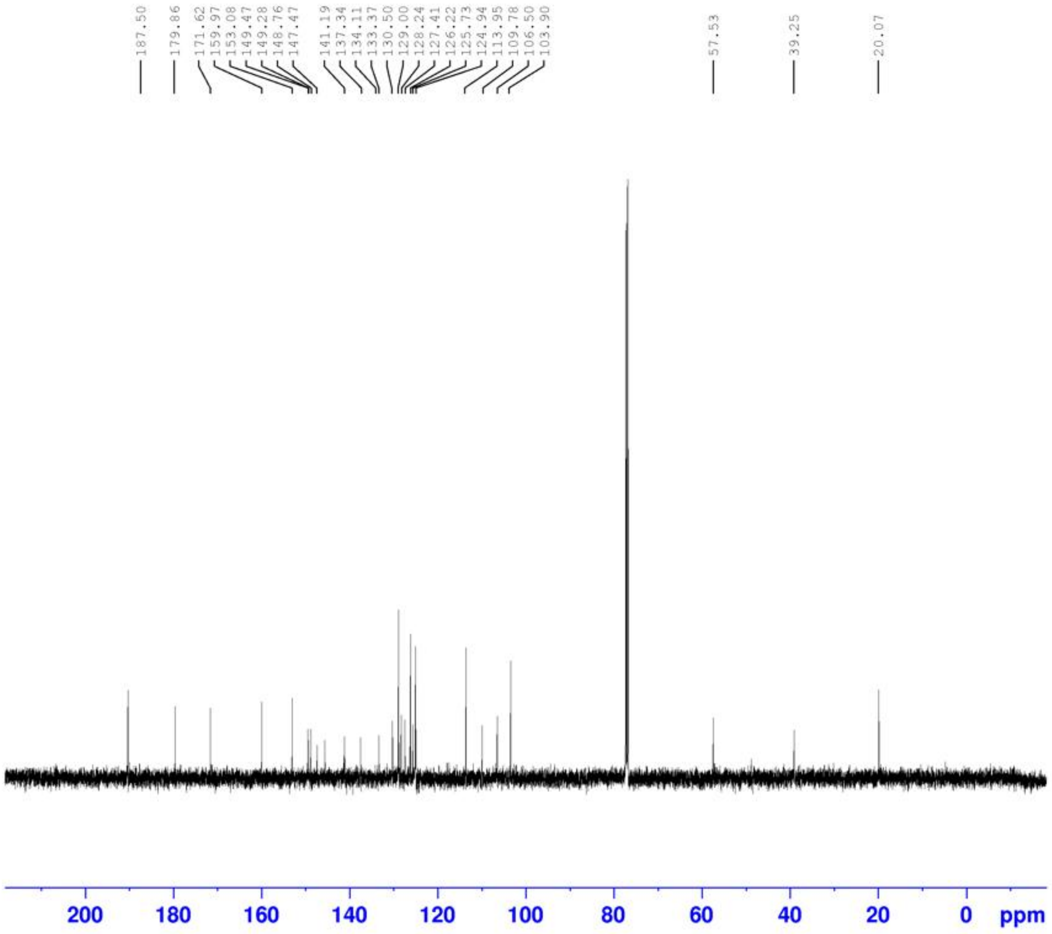

Figure S15: Mass Spectrum of **F-3**

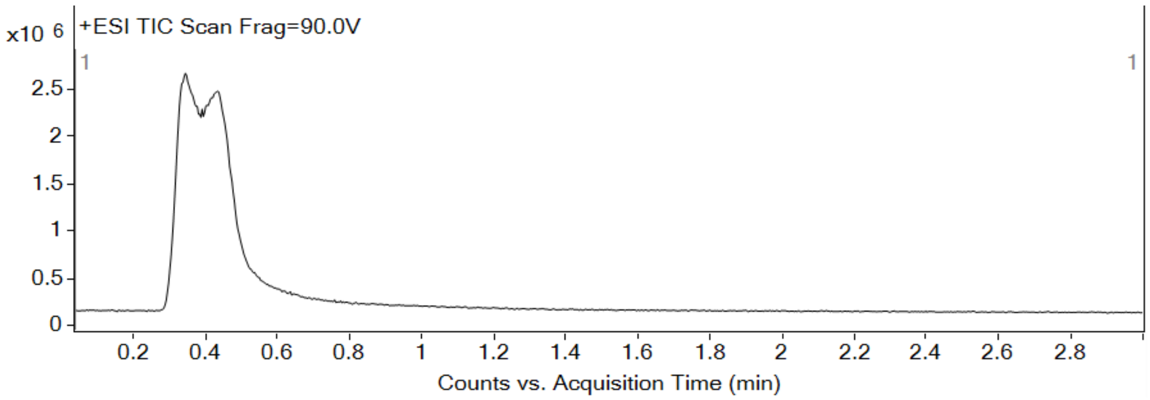

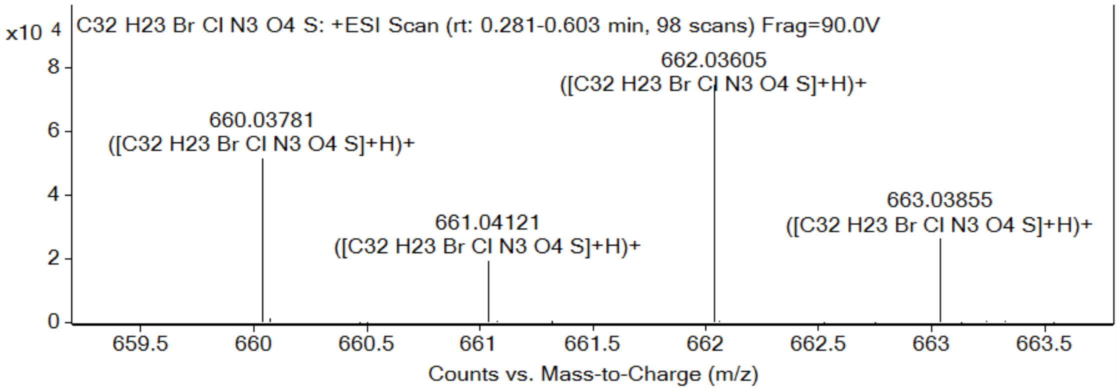

Formula	Best	Mass	Tgt Mass	Diff (ppm)	Ion Species	Score
C32 H23 Br Cl N3 O4 S	TRUE	659,0305	659,02812	3,61	C32 H24 Br Cl N3 O4 S	92,21

Figure S16: ^{1}H NMR Spectrum of **F-4**

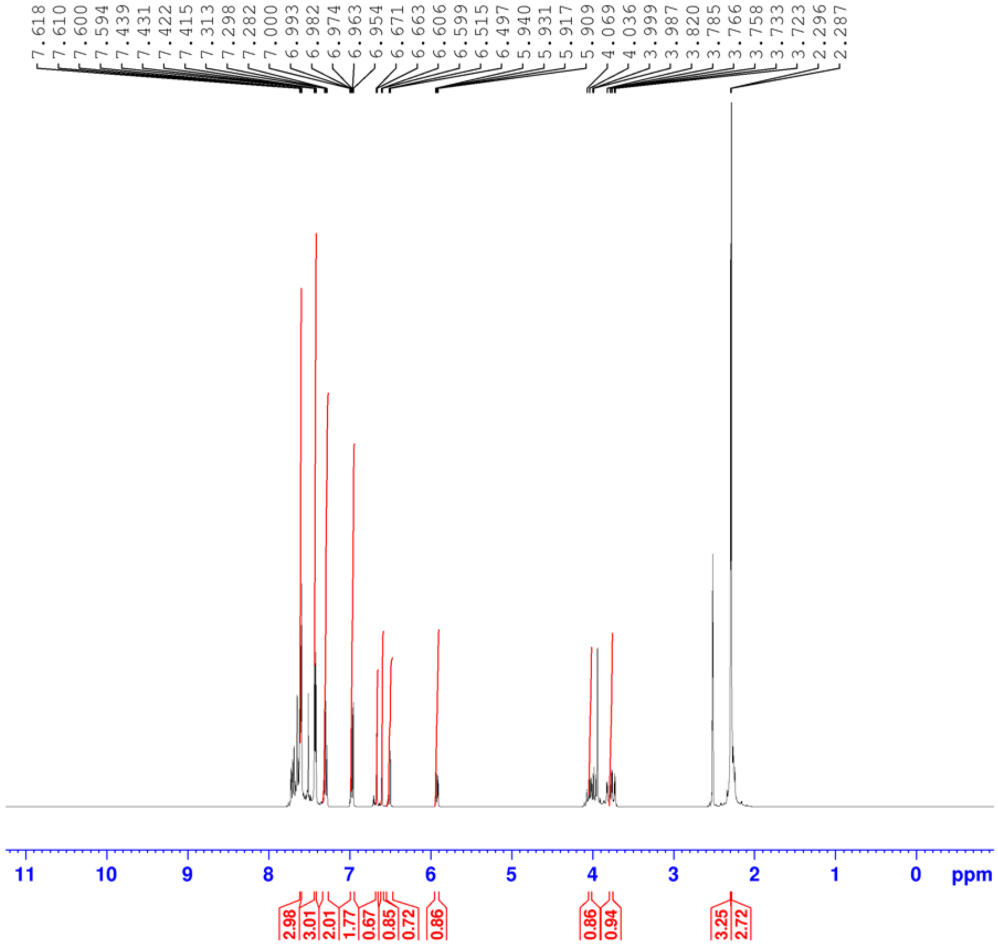

Figure S17: ^{13}C NMR Spectrum of **F-4**

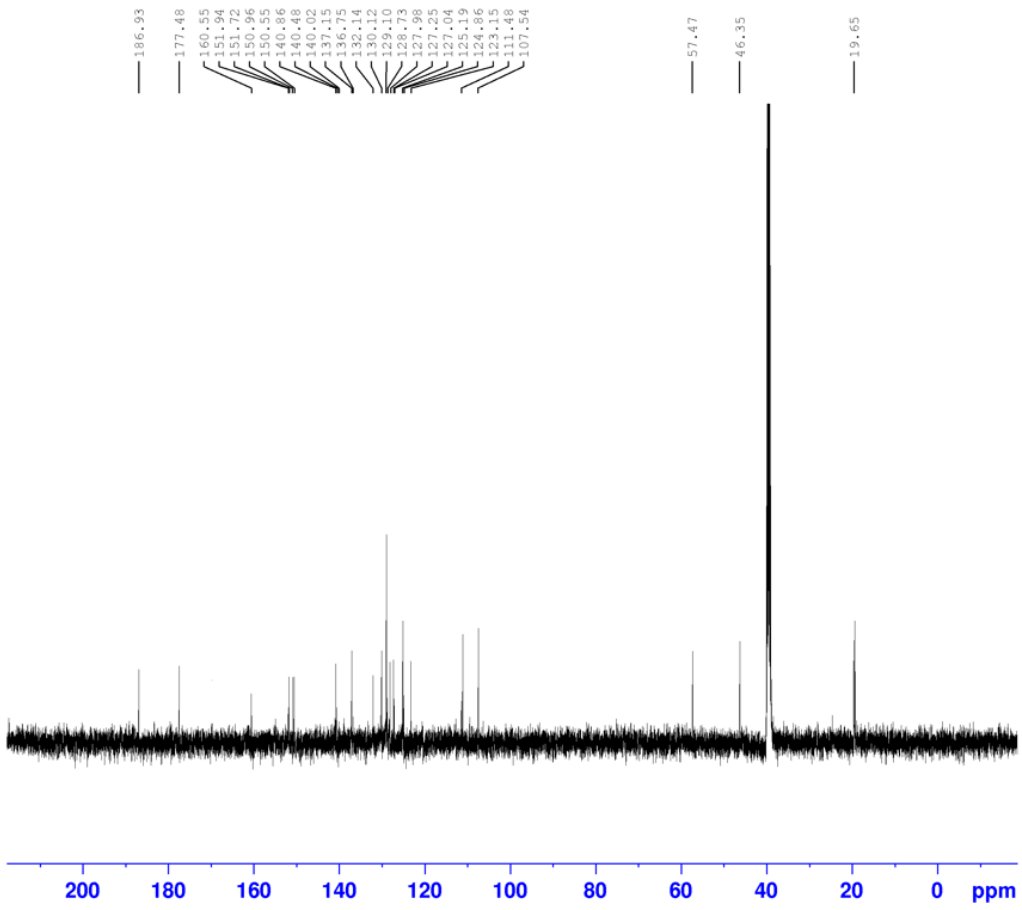

Figure S18: Mass Spectrum of **F-4**

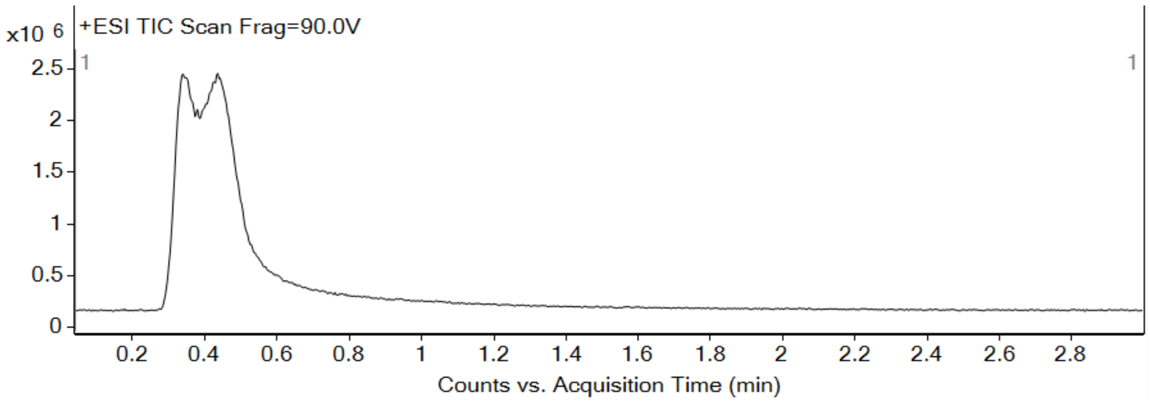

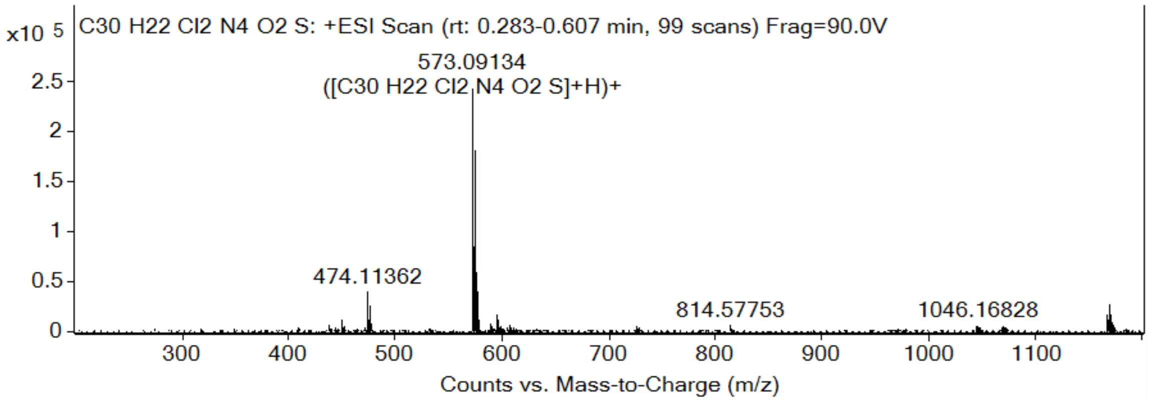

Formula	Best	Mass	Tgt Mass	Diff (ppm)	Ion Species	Score
C30 H22 Cl2 N4 O2 S	TRUE	572,08455	572,08405	0,87	C30 H23 Cl2 N4 O2 S	98,24

Figure S19: ^{1}H NMR Spectrum of **F-5**

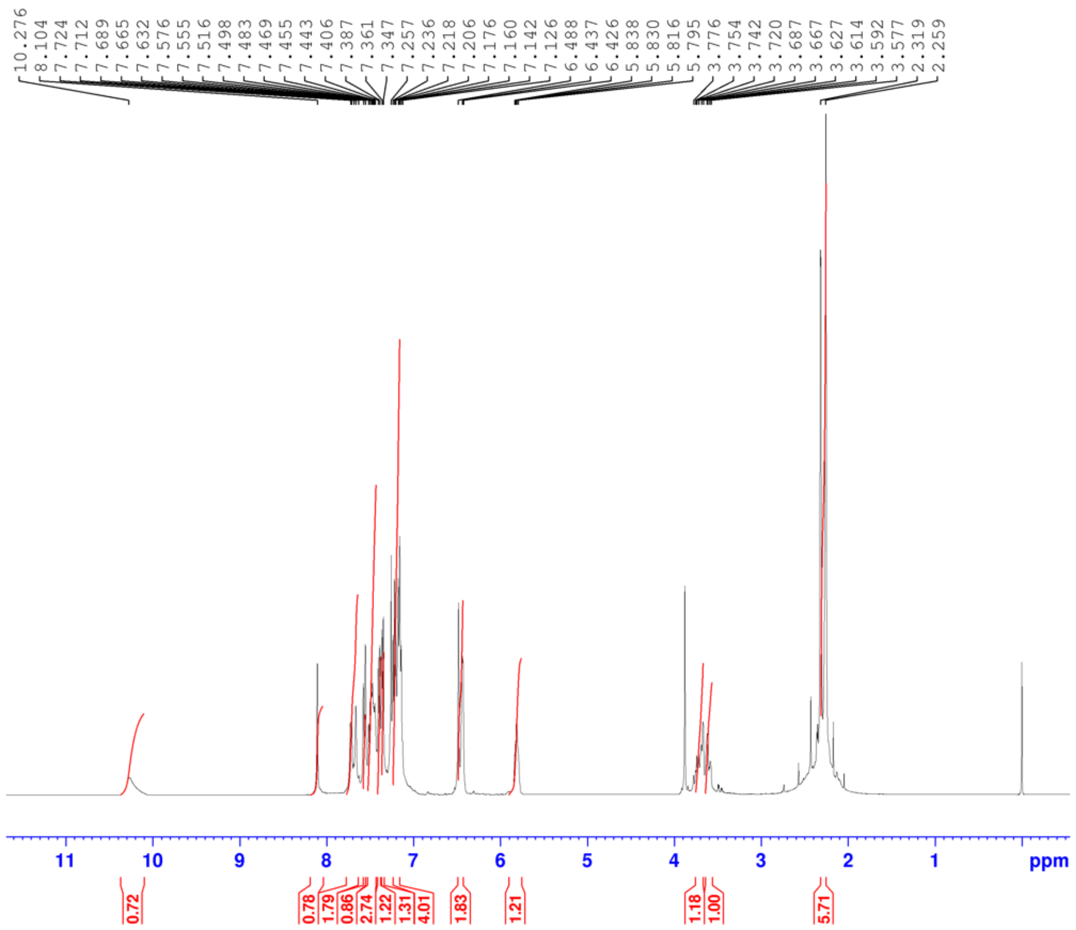

Figure S20: ^{13}C NMR Spectrum of **F-5**

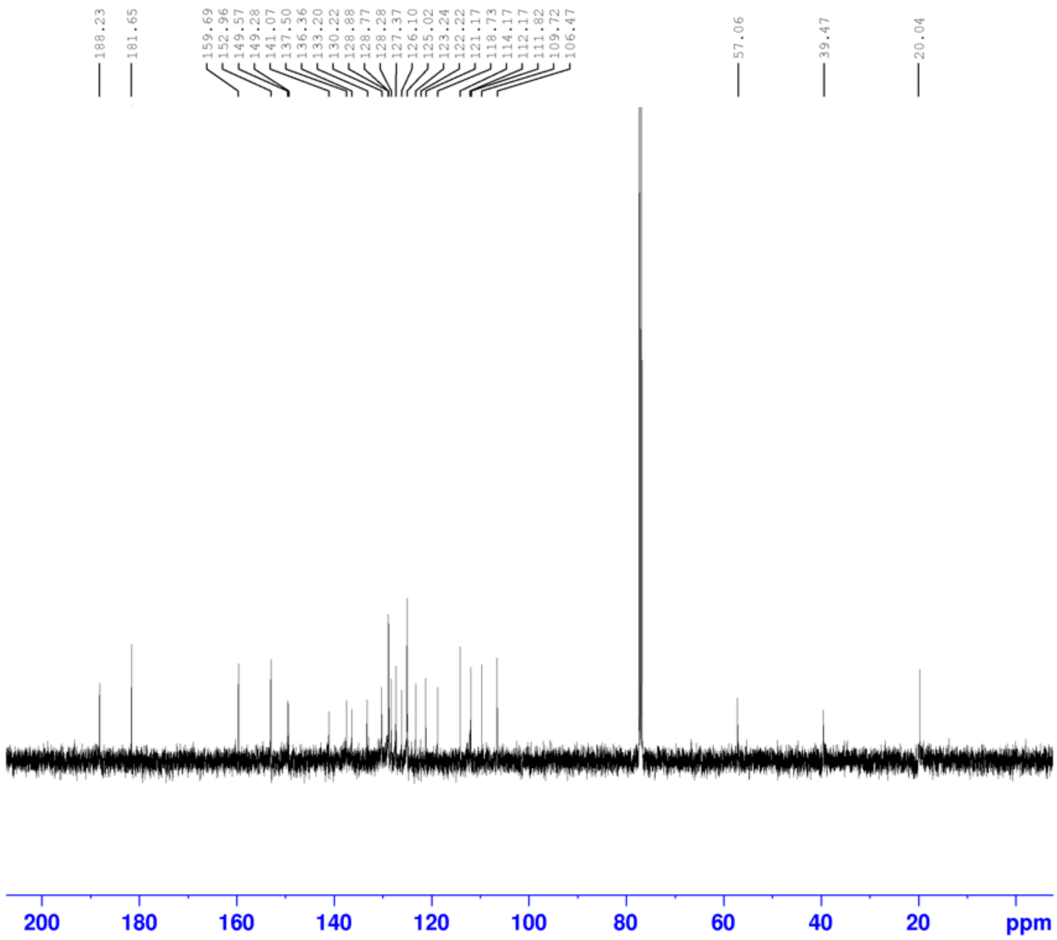

Figure S21: Mass Spectrum of **F-5**

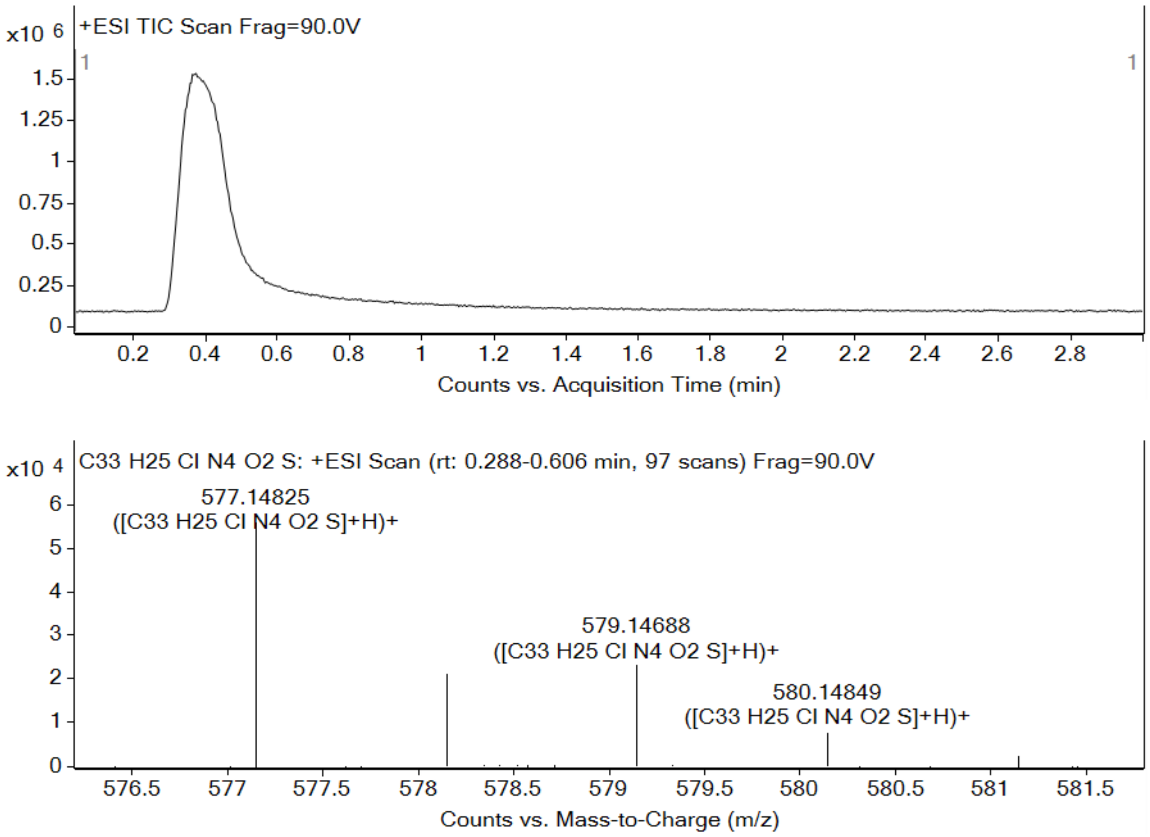

Formula	Best	Mass	Tgt Mass	Diff (ppm)	Ion Species	Score
C33 H25 Cl N4 O2 S	TRUE	576,141	576,13867	4,04	C33 H26 Cl N4 O2 S	91,04
C33 H25 Cl N4 O2 S	TRUE	576,1405	576,13867	3,17	C33 H25 Cl N4 Na O2 S	92,74

Figure S22: ^{1}H NMR Spectrum of **F-6**

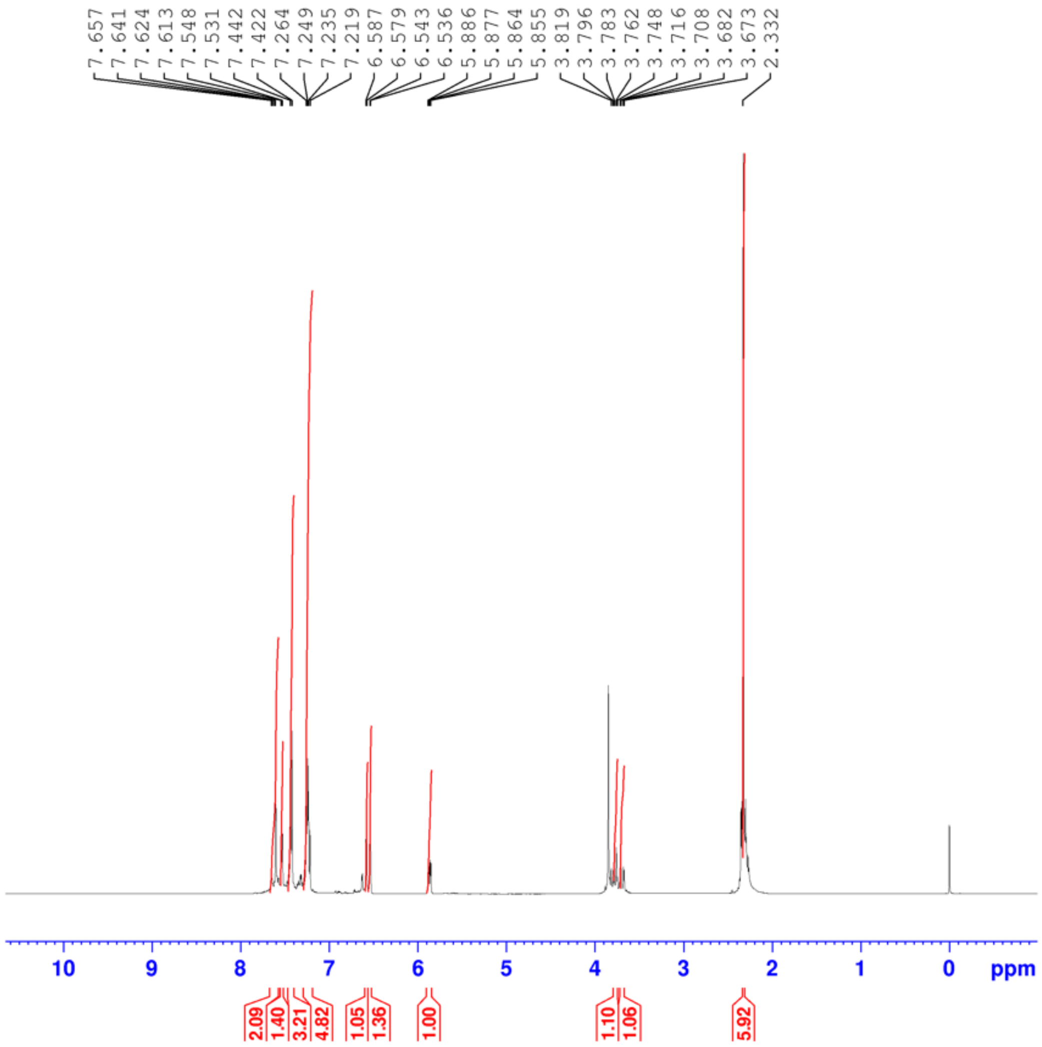

Figure S23: ^{13}C NMR Spectrum of **F-6**

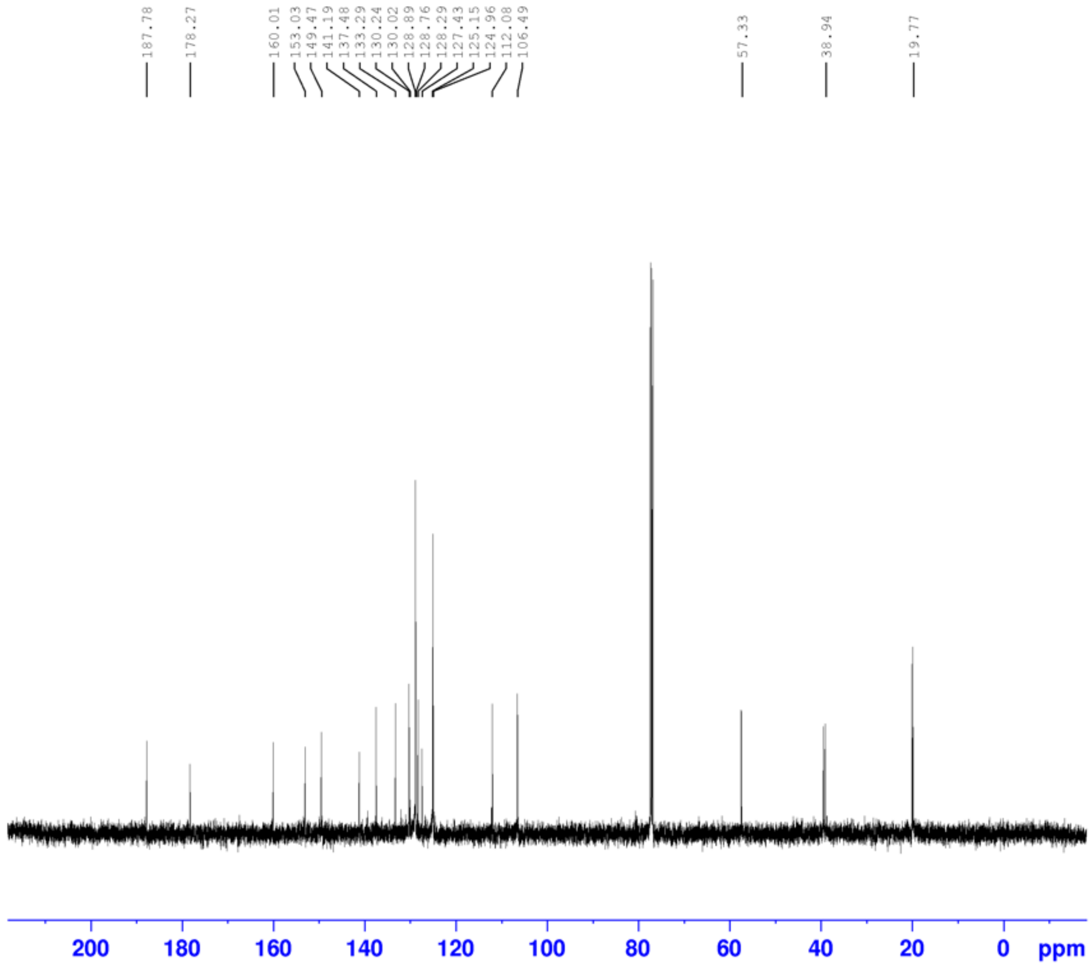

Figure S24: Mass Spectrum of **F-6**

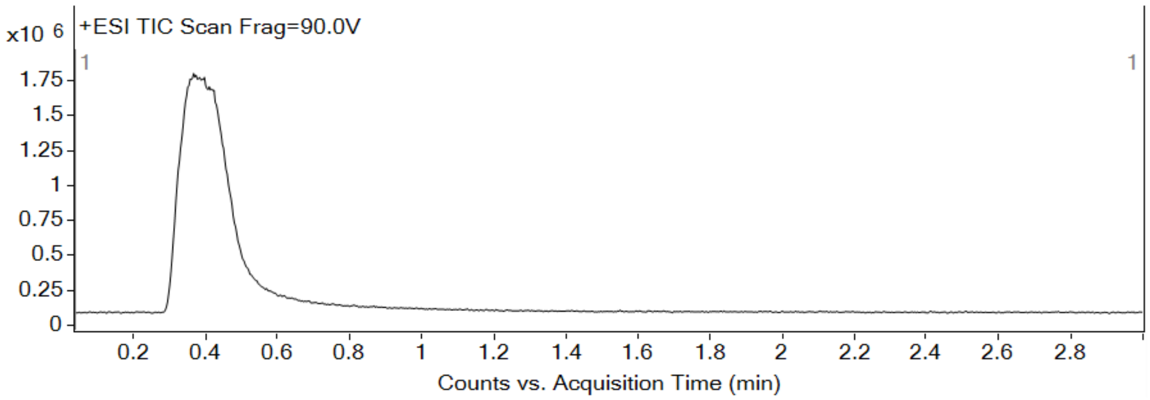

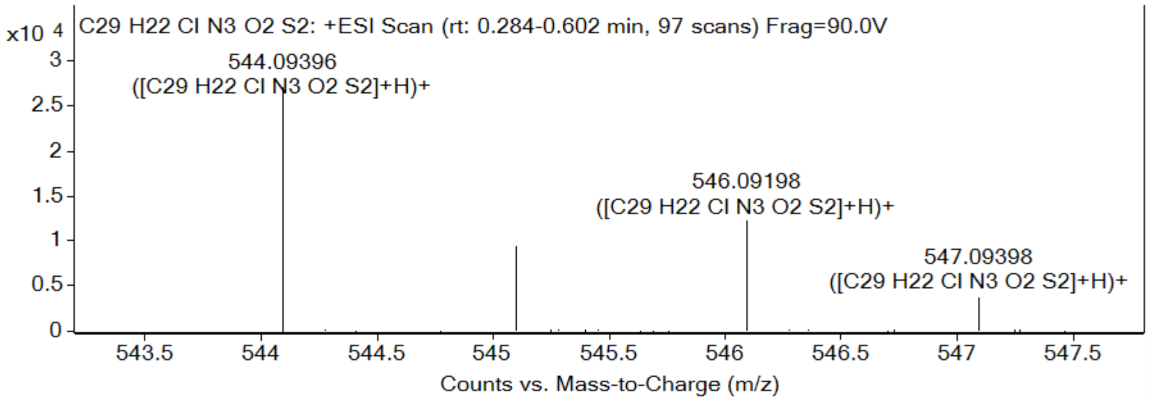

Formula	Best	Mass	Tgt Mass	Diff (ppm)	Ion Species	Score
C29 H22 Cl N3 O2 S2	TRUE	543,08669	543,0842	4,59	C29 H23 Cl N3 O2 S2	89,3

Figure S25: ^{1}H NMR Spectrum of **F-7**

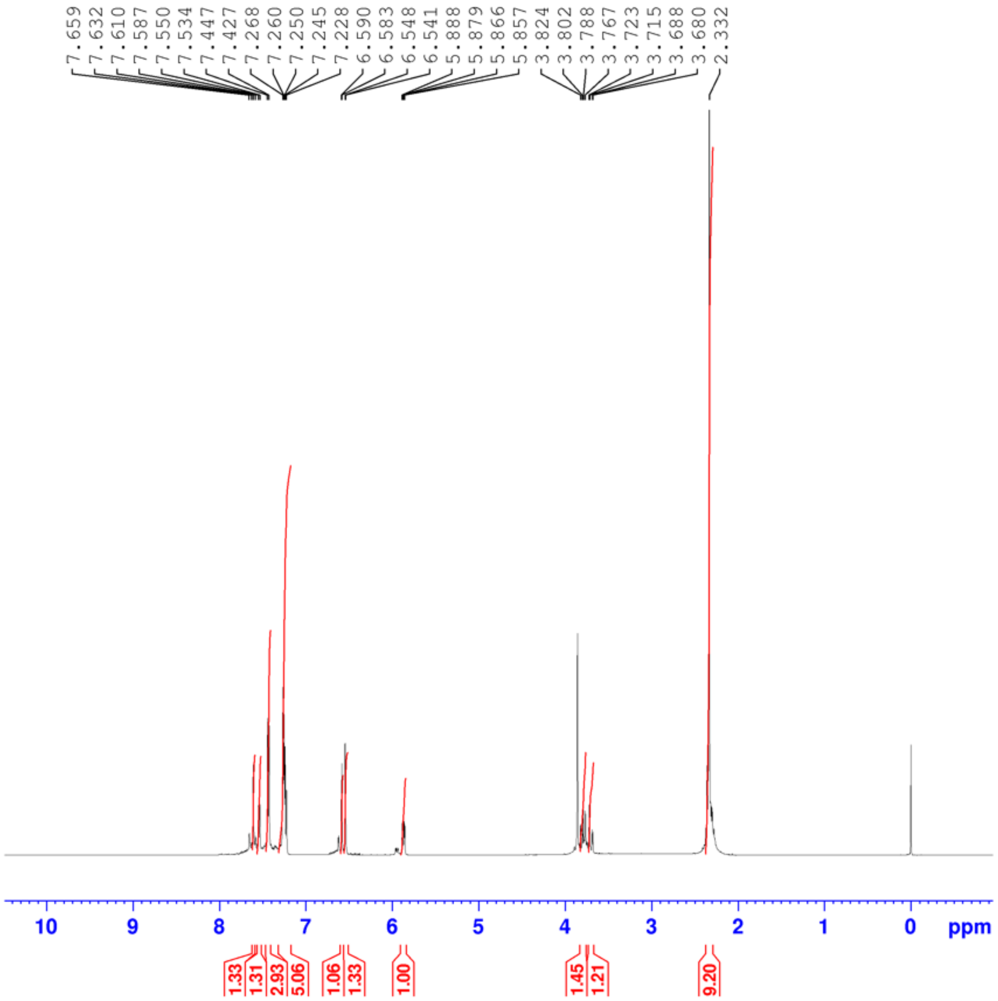

Figure S26: ^{13}C NMR Spectrum of **F-7**

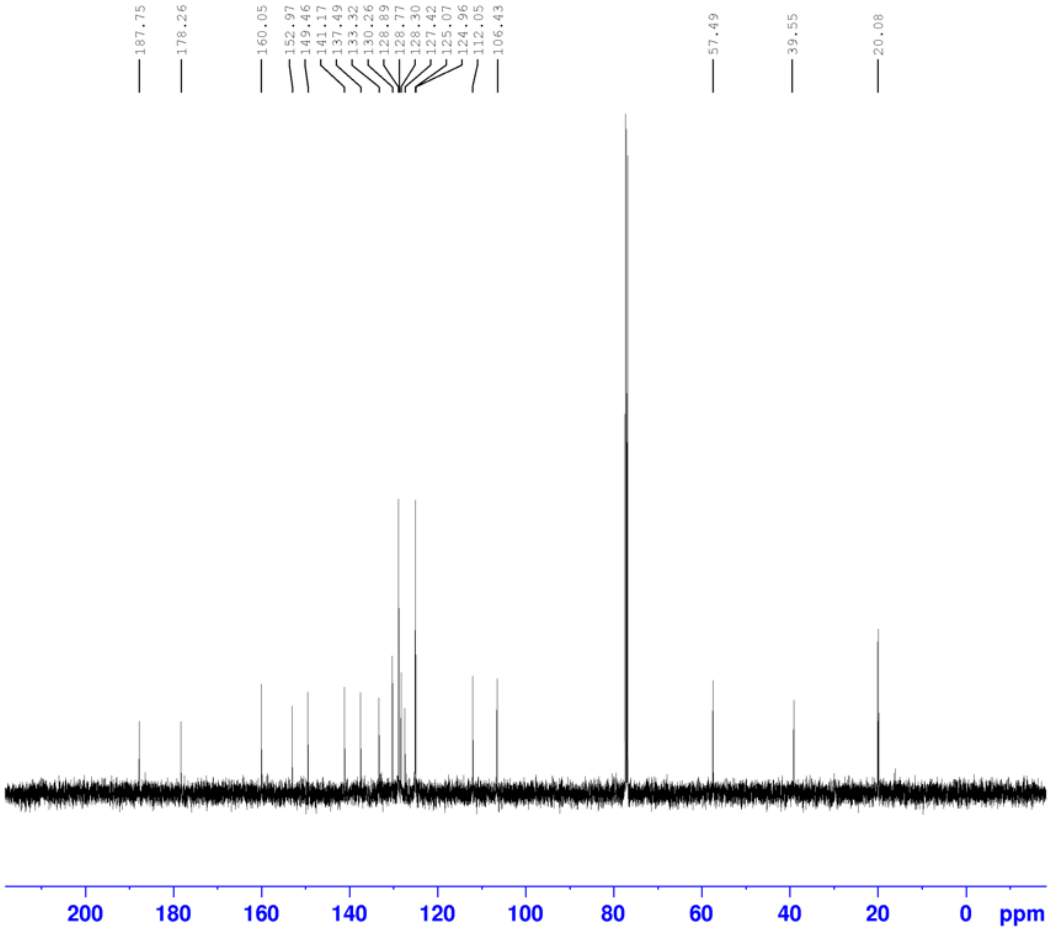

Figure S27: Mass Spectrum of **F-7**

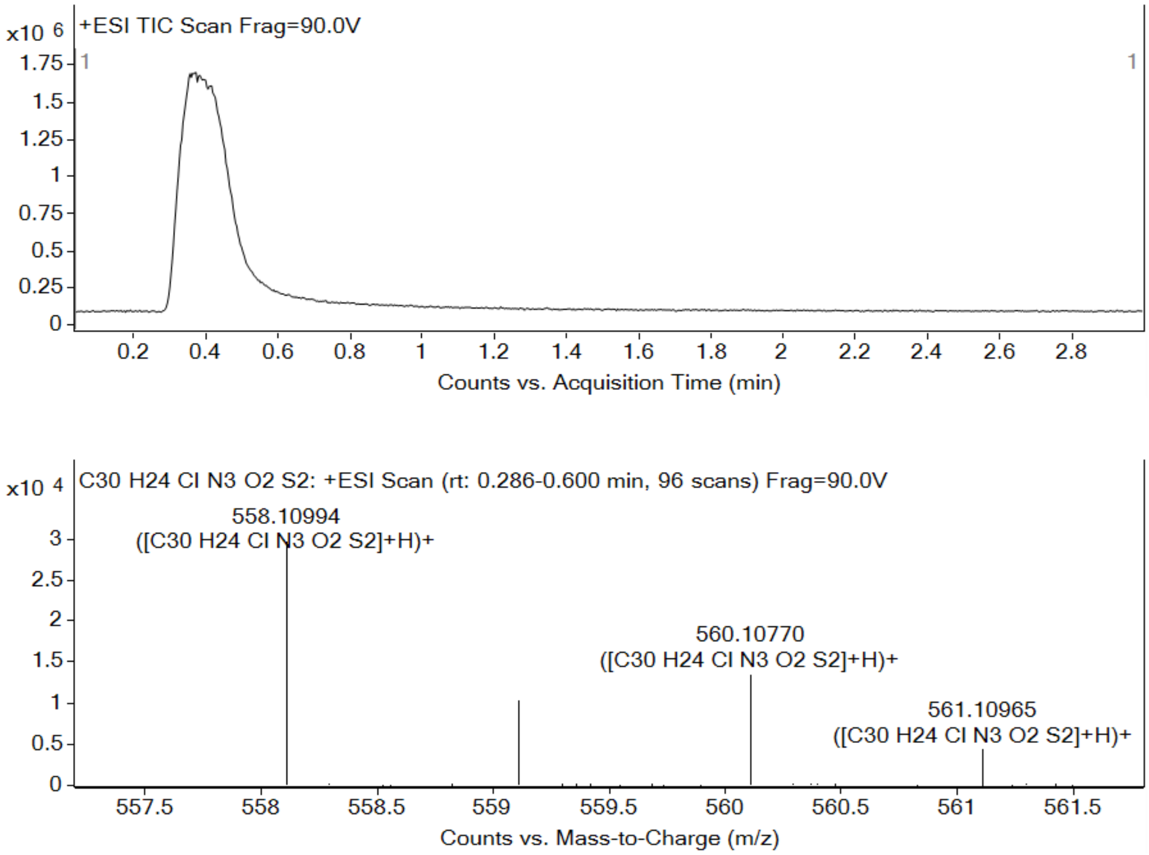

Formula	Best	Mass	Tgt Mass	Diff (ppm)	Ion Species	Score
C30 H24 Cl N3 O2 S2	TRUE	557,10244	557,09985	4,66	C30 H25 Cl N3 O2 S2	88,86
C30 H24 Cl N3 O2 S2	TRUE	557,10202	557,09985	3,9	C30 H24 Cl N3 Na O2 S2	72,78

Figure S28: ^{1}H NMR Spectrum of **F-8**

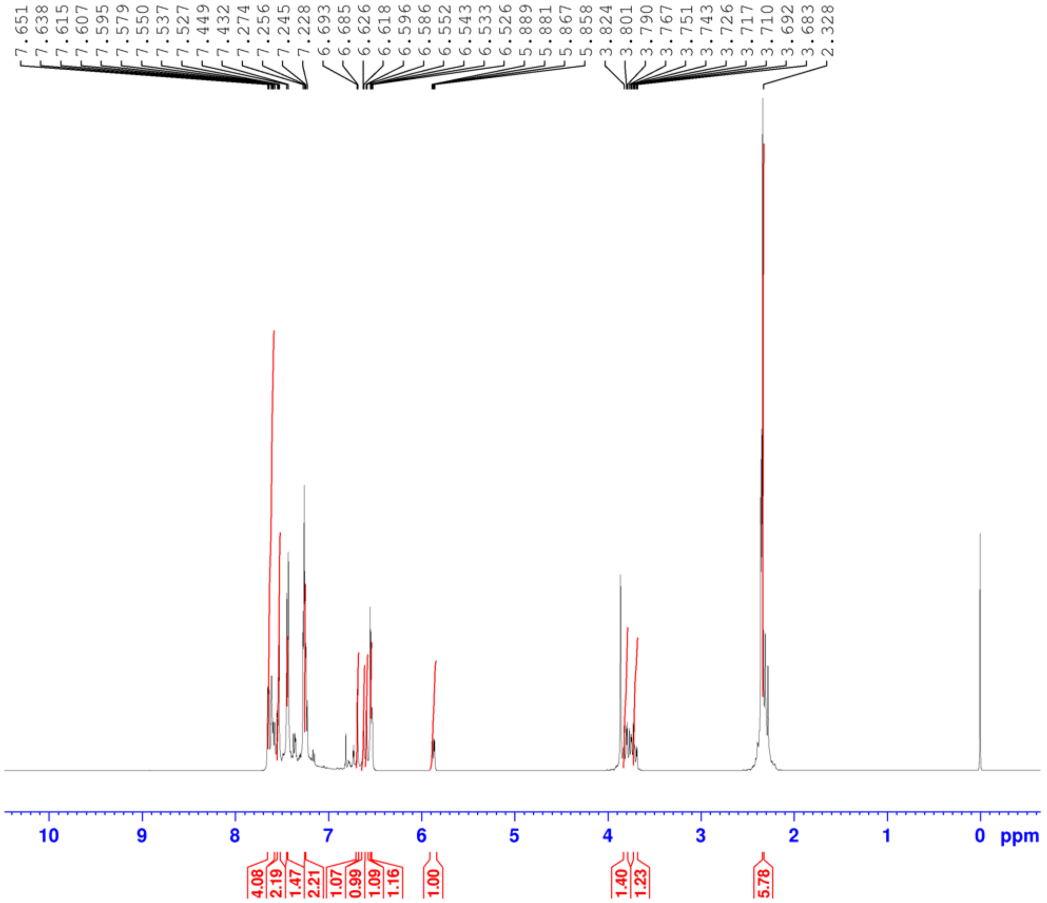

Figure S29. ^{13}C NMR Spectrum of **F-8**

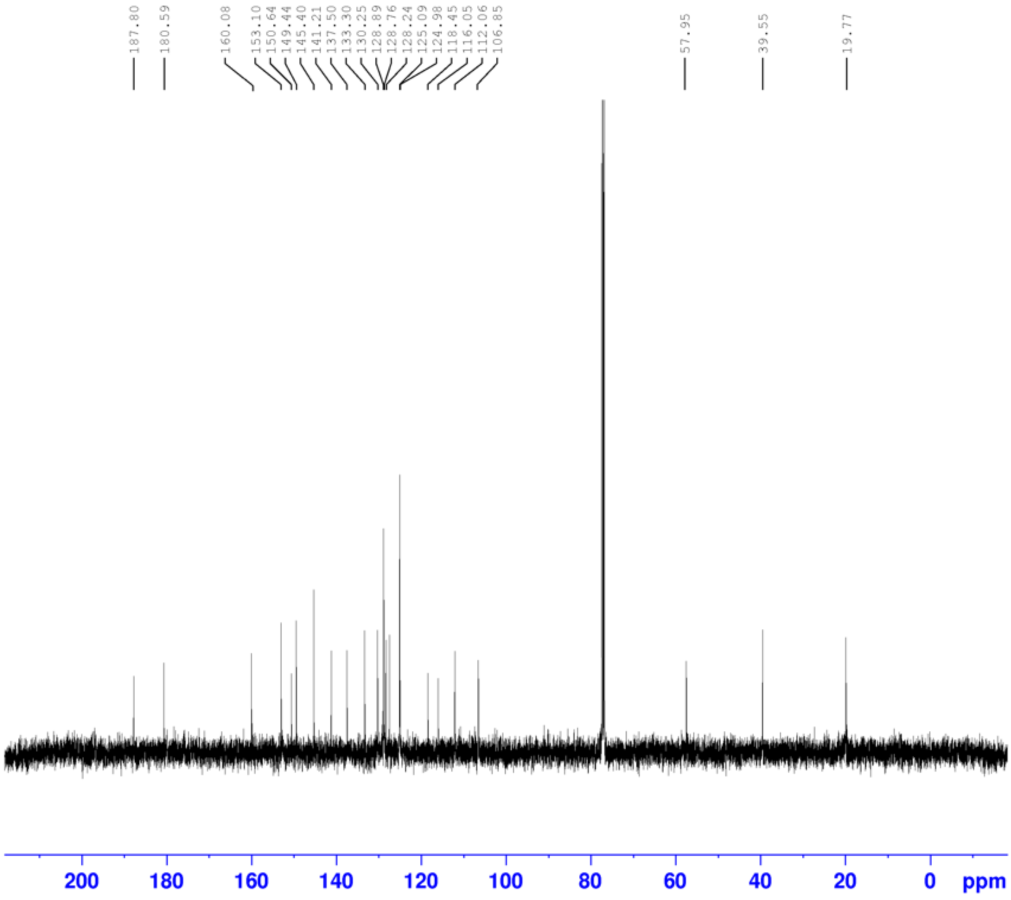

Figure S30. Mass Spectrum of **F-8**

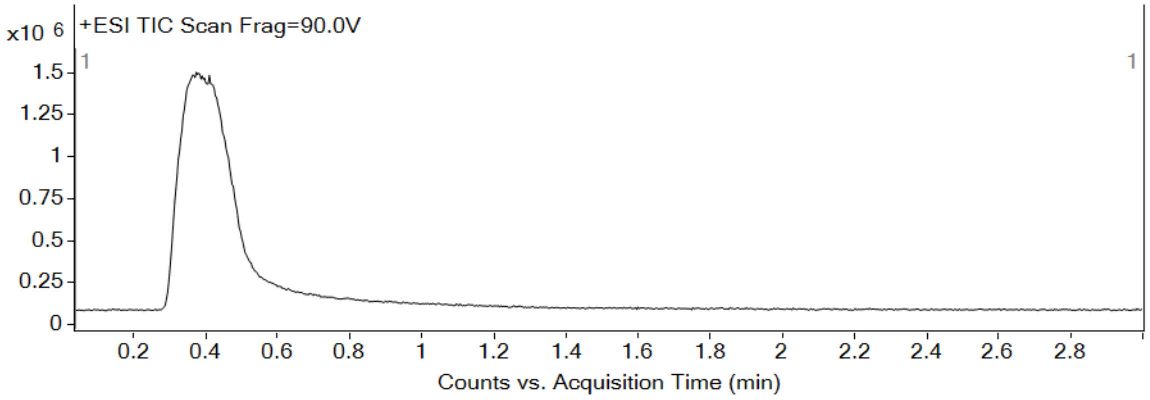

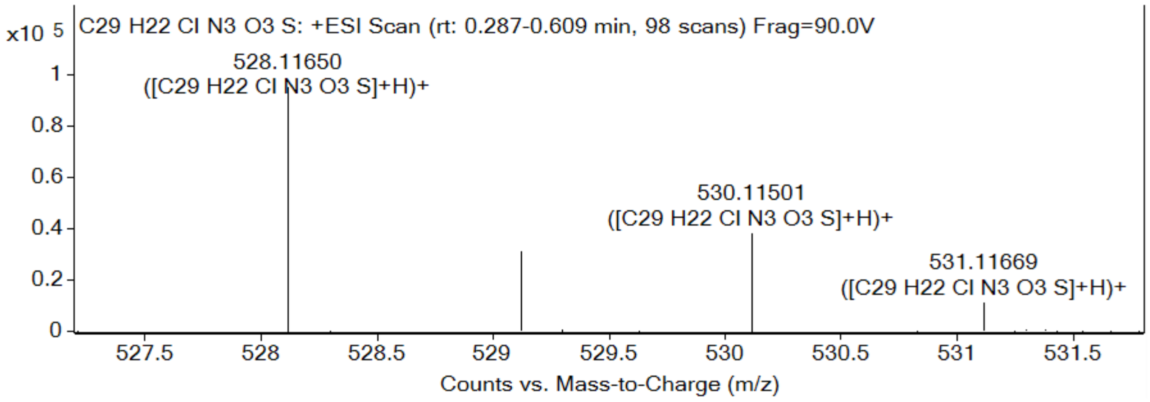

Formula	Best	Mass	Tgt Mass	Diff (ppm)	Ion Species	Score
C29 H22 Cl N3 O3 S	TRUE	527,1093	527,10704	4,28	C29 H23 Cl N3 O3 S	90,65
C29 H22 Cl N3 O3 S	TRUE	527,10875	527,10704	3,24	C29 H22 Cl N3 Na O3 S	93,74

Figure S31: ^{1}H NMR Spectrum of **F-9**

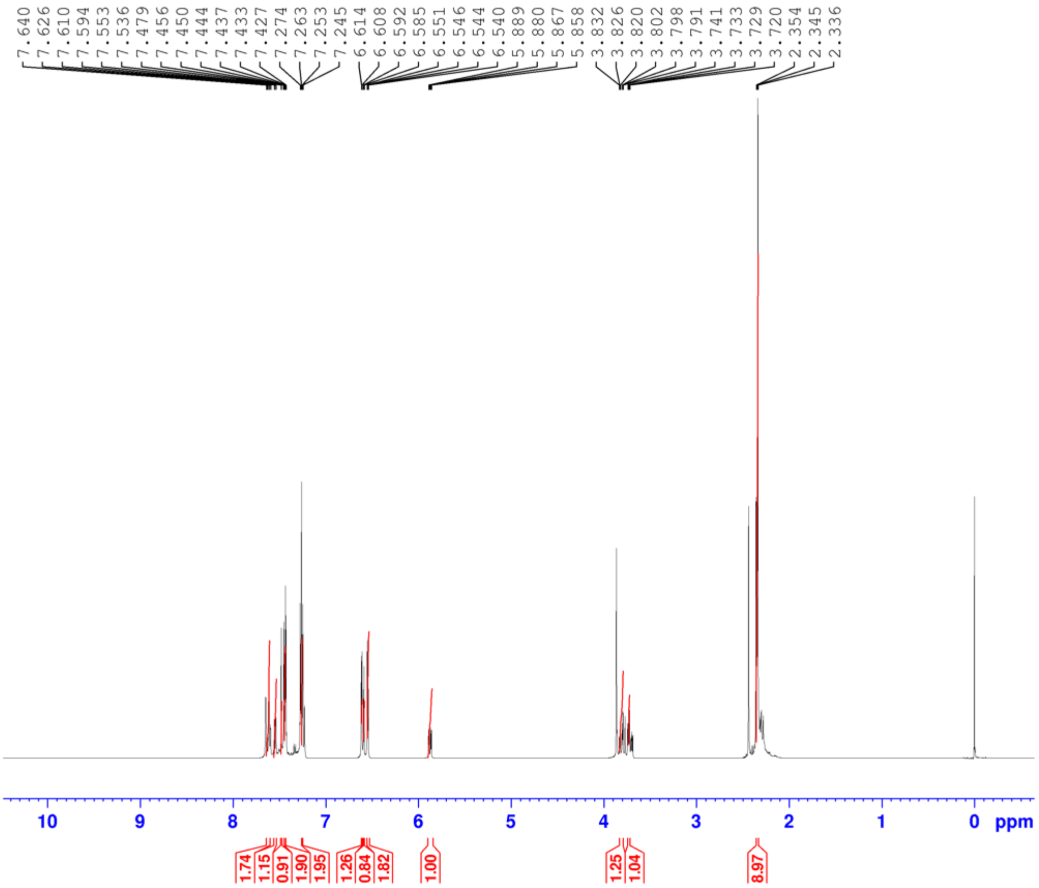

Figure S32: ^{13}C NMR Spectrum of **F-9**

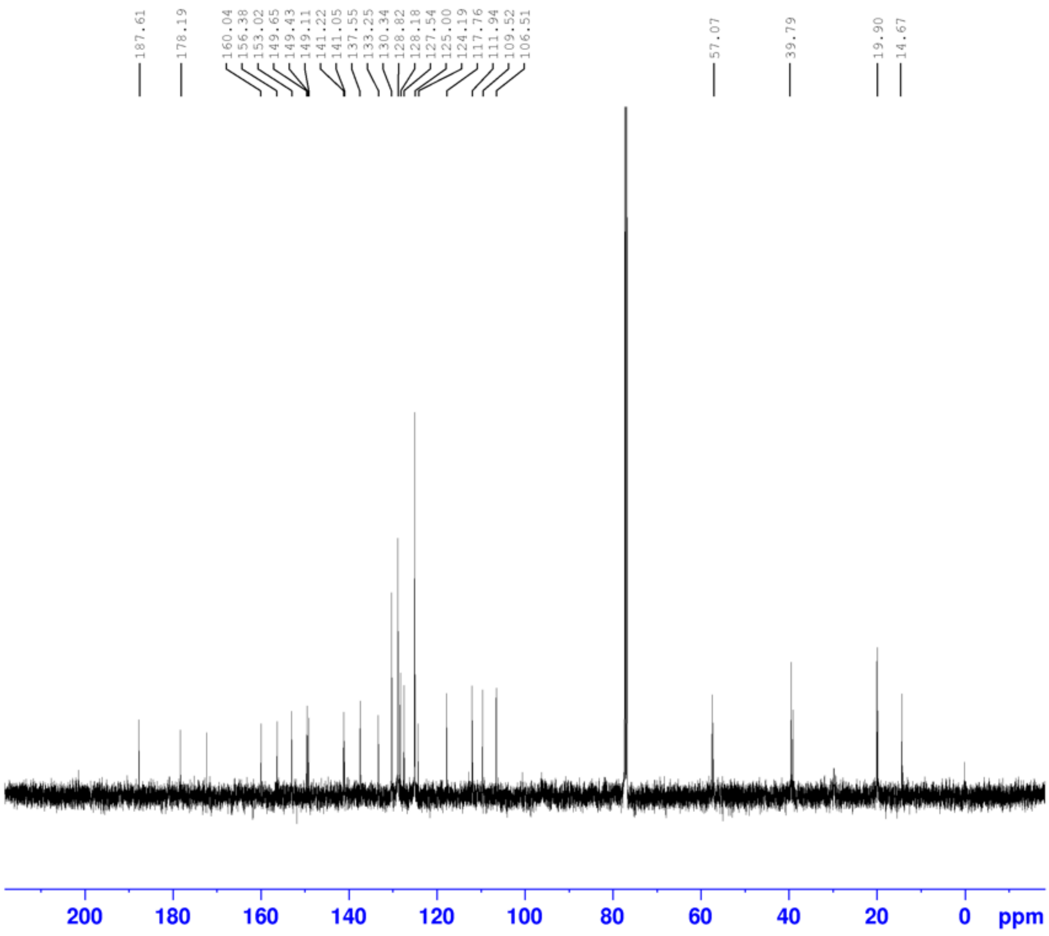

Figure S33: Mass Spectrum of **F-9**

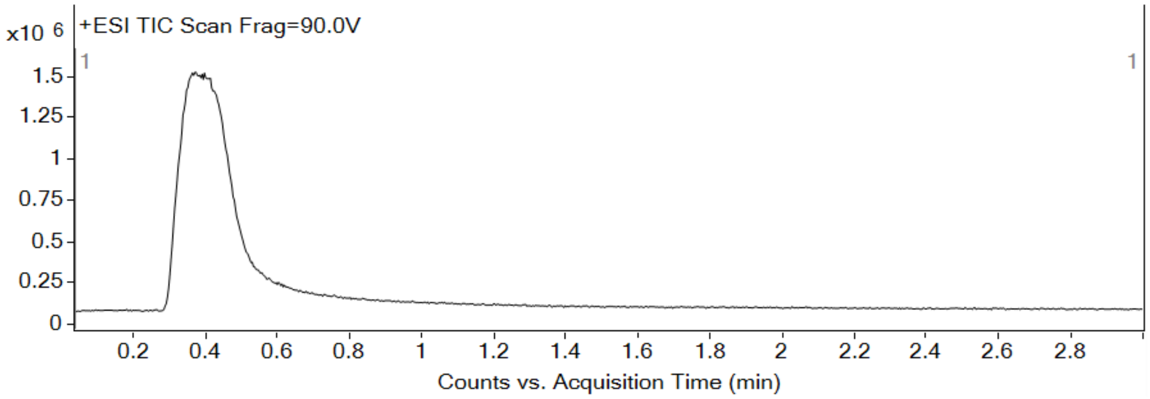

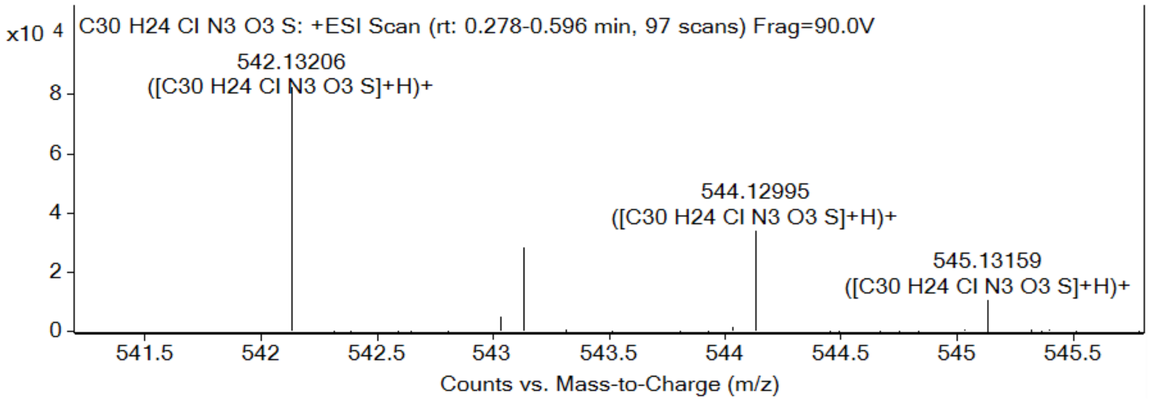

Formula	Best	Mass	Tgt Mass	Diff (ppm)	Ion Species	Score
C30 H24 Cl N3 O3 S	TRUE	541,12447	541,12269	3,29	C30 H25 Cl N3 O3 S	93,64

Figure S34: ^{1}H NMR Spectrum of **F-10**

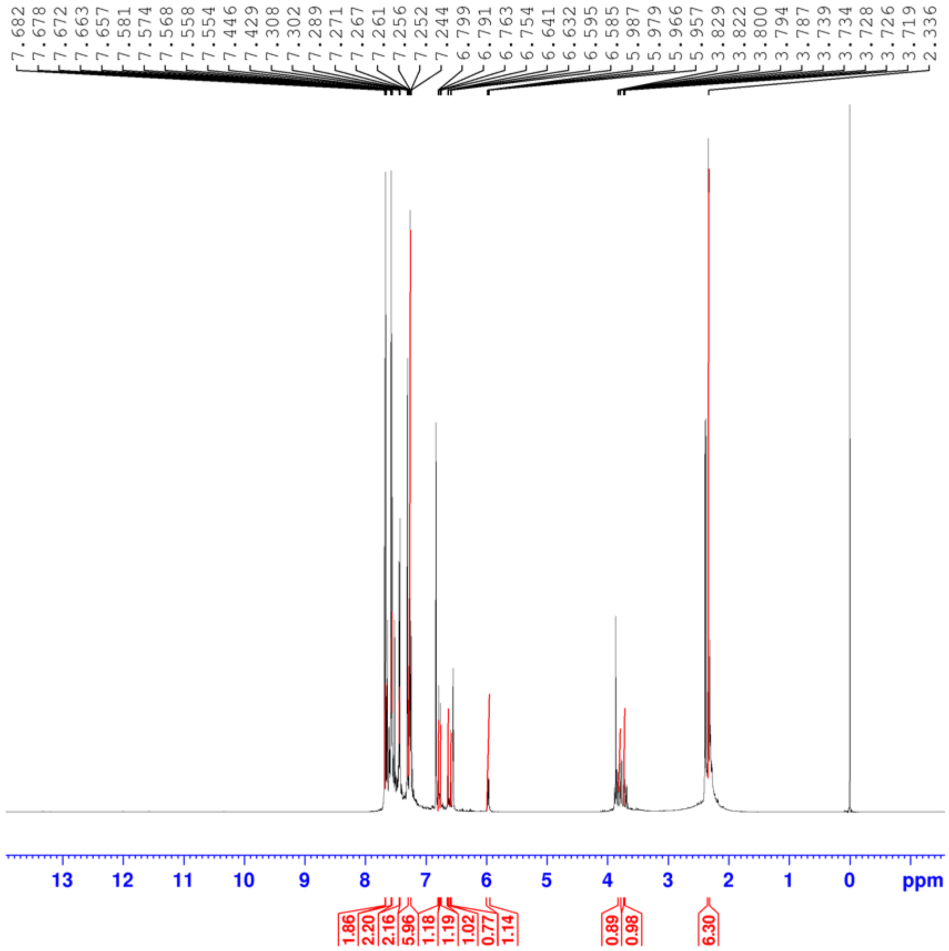

Figure S35: ^{13}C NMR Spectrum of **F-10**

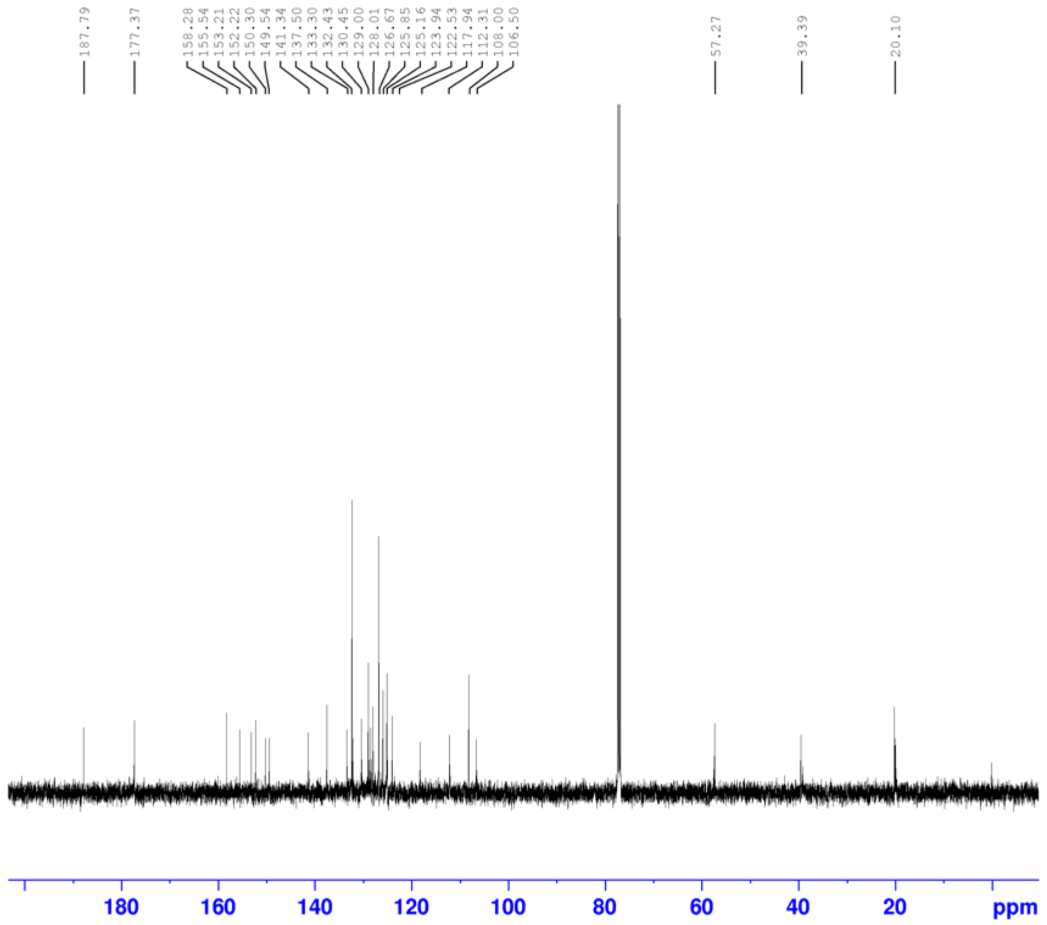

Figure S36: Mass Spectrum of **F-10**

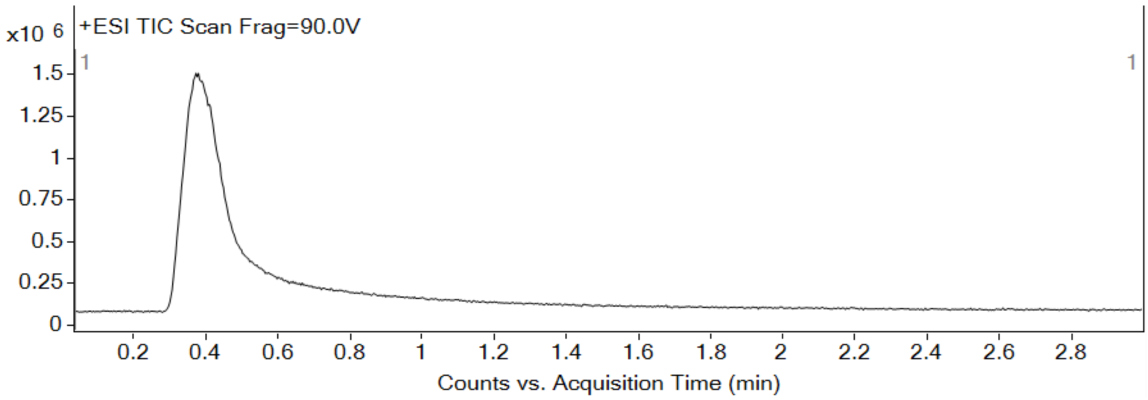

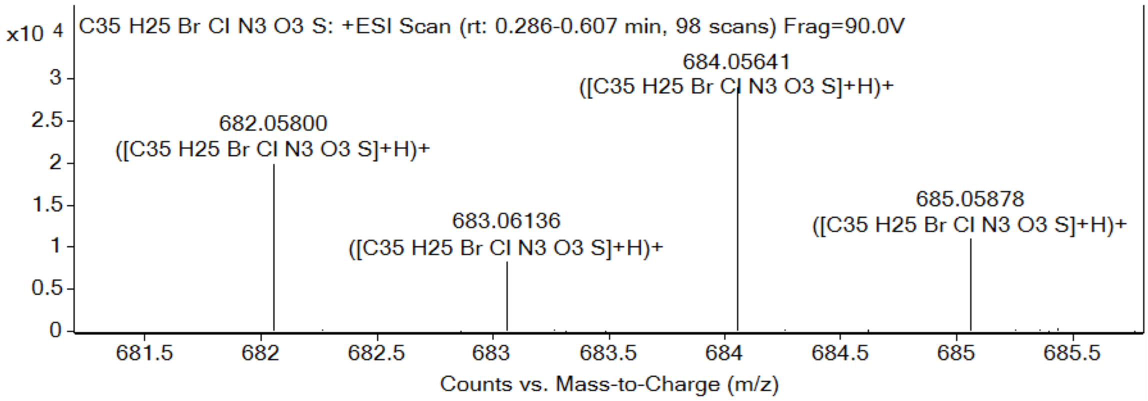

Formula	Best	Mass	Tgt Mass	Diff (ppm)	Ion Species	Score
C35 H25 Br Cl N3 O3 S	TRUE	681,05073	681,04885	2,75	C35 H26 Br Cl N3 O3 S	95,07

Figure S37: ^{1}H NMR Spectrum of **F-11**

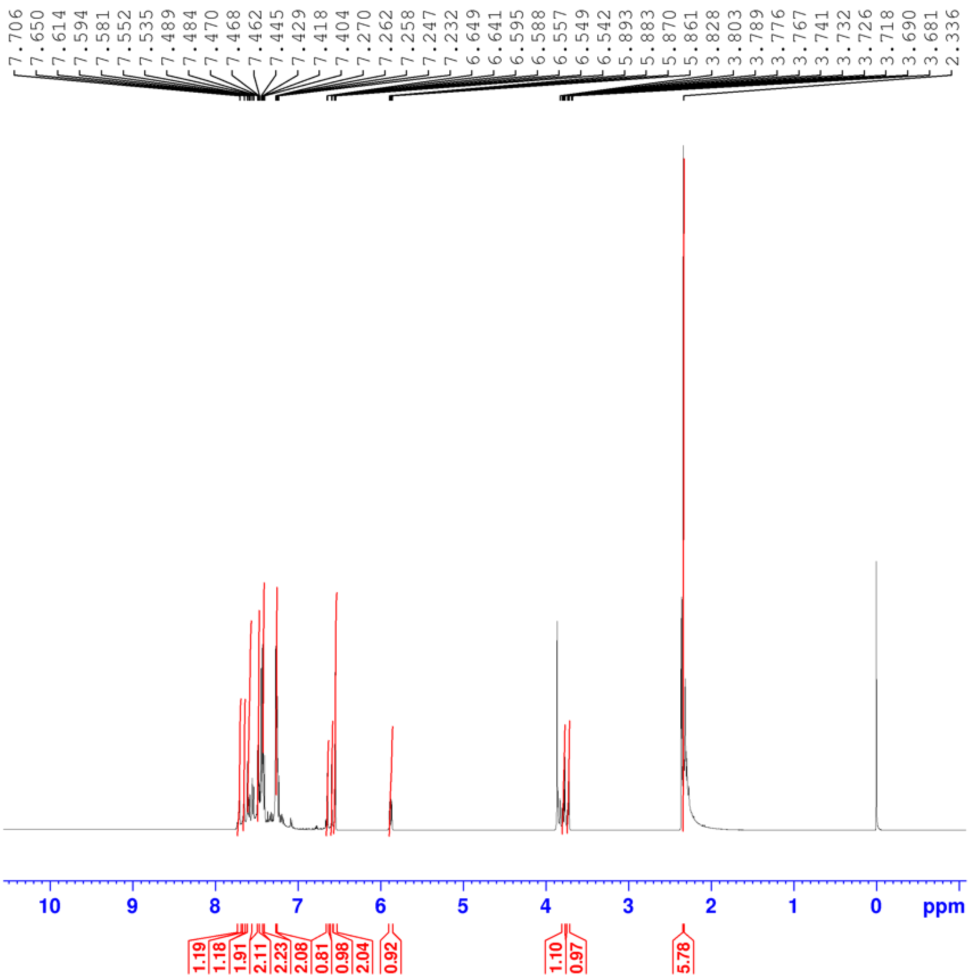

Figure S38: ^{13}C NMR Spectrum of **F-11**

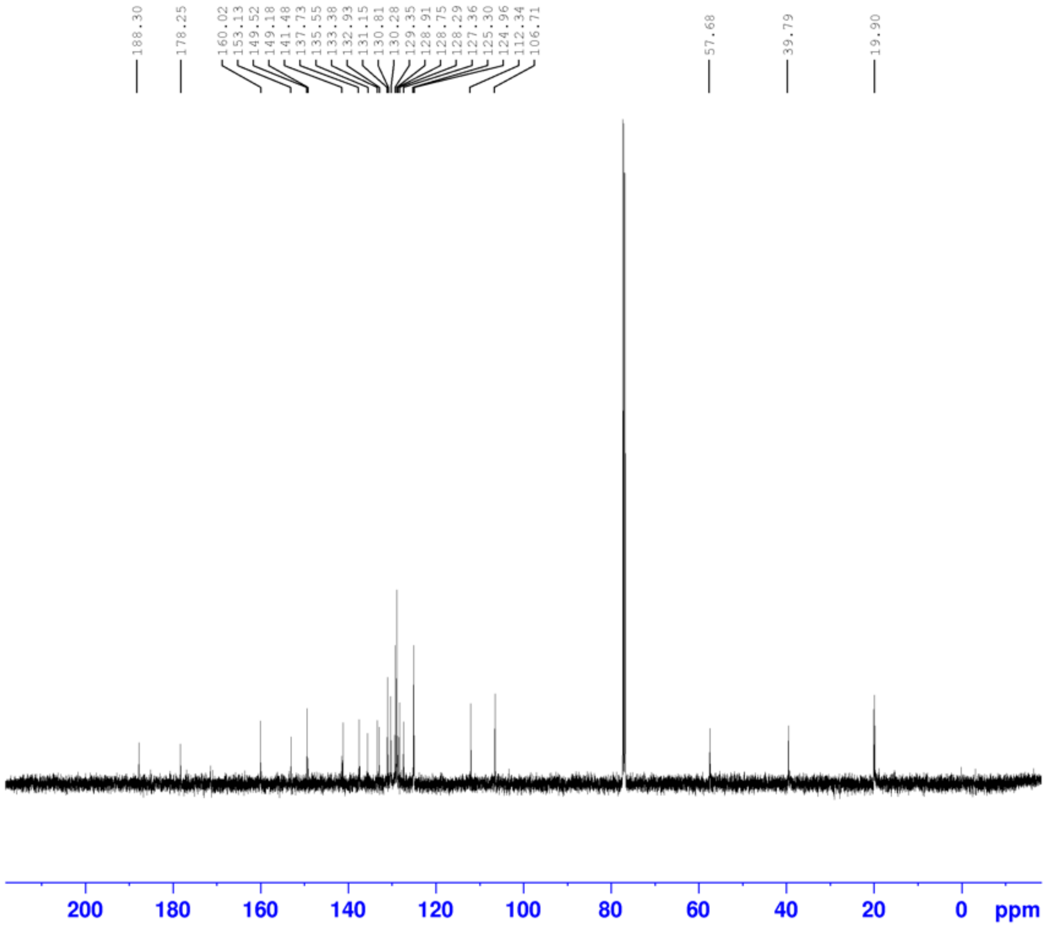

Figure S39: Mass Spectrum of **F-11**

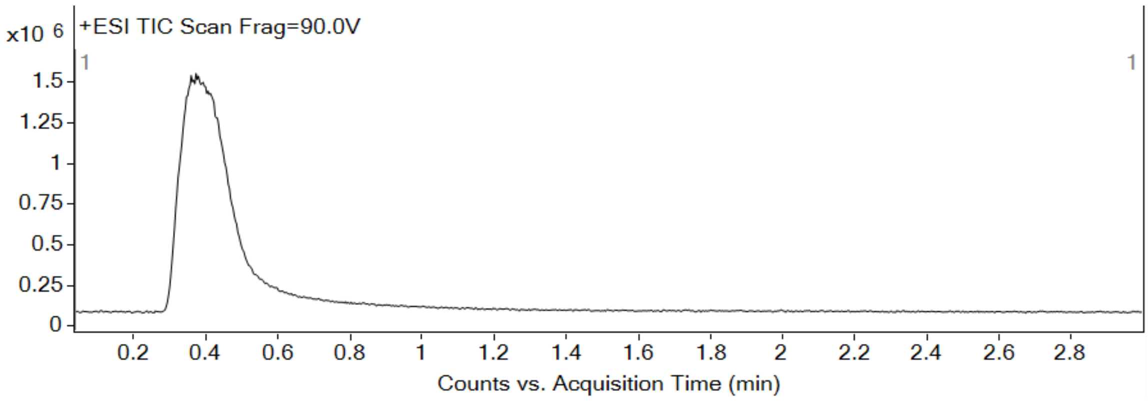

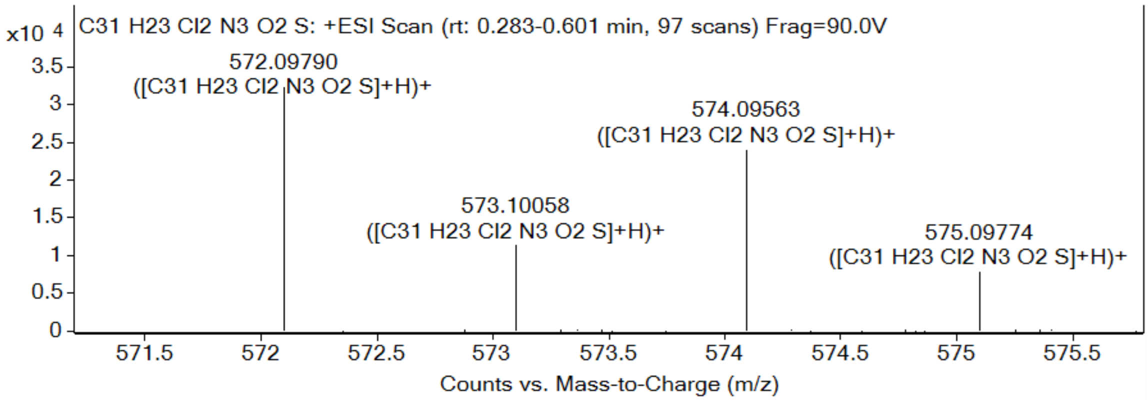

Formula	Best	Mass	Tgt Mass	Diff (ppm)	Ion Species	Score
C31 H23 Cl2 N3 O2 S	TRUE	571,09048	571,0888	2,94	C31 H24 Cl2 N3 O2 S	95,03
C31 H23 Cl2 N3 O2 S	TRUE	571,09076	571,0888	3,42	C31 H23 Cl2 N3 Na O2 S	92,99
